# Characterization and identification of lysine crotonylation sites based on machine learning method on both plant and mammalian

**DOI:** 10.1038/s41598-020-77173-0

**Published:** 2020-11-24

**Authors:** Rulan Wang, Zhuo Wang, Hongfei Wang, Yuxuan Pang, Tzong-Yi Lee

**Affiliations:** 1 School of Science and Engineering, The Chinese University of Hong Kong (Shenzhen), Shenzhen, 518172 Guangdong People’s Republic of China; 2 Warshel Institute for Computational Biology, The Chinese University of Hong Kong (Shenzhen), Shenzhen, 518172 Guangdong People’s Republic of China; 3 School of Life Sciences, University of Science and Technology of China, Hefei, 230026 Anhui People’s Republic of China; 4 Department of Orthopaedics and Traumatology, The University of Hong Kong, Pok Fu Lam, Hong Kong; 5 School of Life and Health Sciences, The Chinese University of Hong Kong (Shenzhen), Shenzhen, 518172 Guangdong People’s Republic of China

**Keywords:** Protein sequence analyses, Machine learning

## Abstract

Lysine crotonylation (Kcr) is a type of protein post-translational modification (PTM), which plays important roles in a variety of cellular regulation and processes. Several methods have been proposed for the identification of crotonylation. However, most of these methods can predict efficiently only on histone or non-histone protein. Therefore, this work aims to give a more balanced performance in different species, here plant (non-histone) and mammalian (histone) are involved. SVM (support vector machine) and RF (random forest) were employed in this study. According to the results of cross-validations, the RF classifier based on EGAAC attribute achieved the best predictive performance which performs competitively good as existed methods, meanwhile more robust when dealing with imbalanced datasets. Moreover, an independent test was carried out, which compared the performance of this study and existed methods based on the same features or the same classifier. The classifiers of SVM and RF could achieve best performances with 92% sensitivity, 88% specificity, 90% accuracy, and an MCC of 0.80 in the mammalian dataset, and 77% sensitivity, 83% specificity, 70% accuracy and 0.54 MCC in a relatively small dataset of mammalian and a large-scaled plant dataset respectively. Moreover, a cross-species independent testing was also carried out in this study, which has proved the species diversity in plant and mammalian.

## Introduction

Post-translational modifications(PTMs) modulate the activity of most eukaryote proteins^1^, which play pivotal roles in numerous biological processes by modulating regulation of protein function and cellular processes^2^ such as histone acetylation, which plays a significant role in mammalian DNA repair^3^. Sumoylation was found on transcription factors with greatly increased frequencies, which shows it has a large impact on the transcription of protein^4^. Signaling pathways^5^, protein-protein interactions^6,7^, apoptosis^8^, cell death^9^, and metabolic pathways^10,11^ are all affected by various kinds of PTMs. Owing to the importance of PTMs, several datasets of annotated PTMs of various types have been released in decades, such as emerging *S*-nitrosylation, *S*-glutathionylation and succinylation^12^, which provided enough resources for investigation. Beside those earlier-discovered PTMs, crotonylation is a recently discovered one, which was originally found in somatic and mouse male germ cell and enriched on sex chromosomes^13^, and of significant importance in regulating various of biological processes. The abundance of MS-verified crotonylated peptides enabled the investigation of substrate site specificity of crotonylation sites through sequence-based attributes^14^. In 2017, Ju and He have proposed an SVM-based method by using attribute CKSAAP for this prediction, and a tool named CKSAAP_CrotSite was developed that time^15^; also in 2017, Wang has proposed another method based on ensemble RF, which employed the attribute of pseudo-AAC^16^. In 2018, 5995 sites on 2120 proteins have first been extracted and released by Liu et al.^17^ and provided more experimental-verified crotonylated samples in plant *Carica papaya* L., which filled in the gaps of lacking samples in computational analysis of crotonylation. Based on these *Carica papaya* L. data, Zhao et al. has carried a prediction on the large dataset, in which deep learning method has been involved^18^. However, these prediction processes are of certain limitations. First, some predictions are based on small dataset with protein number no more than 400 proteins, which can not be convincing. Meanwhile, the prediction based on *Carica papaya* L. is of enough quantity, but owing to the imbalance of dataset in positive and negative sample number, the result is biased, which can not be efficient in most of the real case. Hence, an overall investigation on different species with enough quantity of data in a proper classification method needs to be addressed. In this study, we have gathered both plant and mammalian samples, and employed classical machine learning methods for the prediction of crotonylation in both plant and mammalian datasets, which is the first evaluation on imbalanced cases of different species.

## Result

### Substrate site signatures of lysine crotonylation

**Figure 1:**
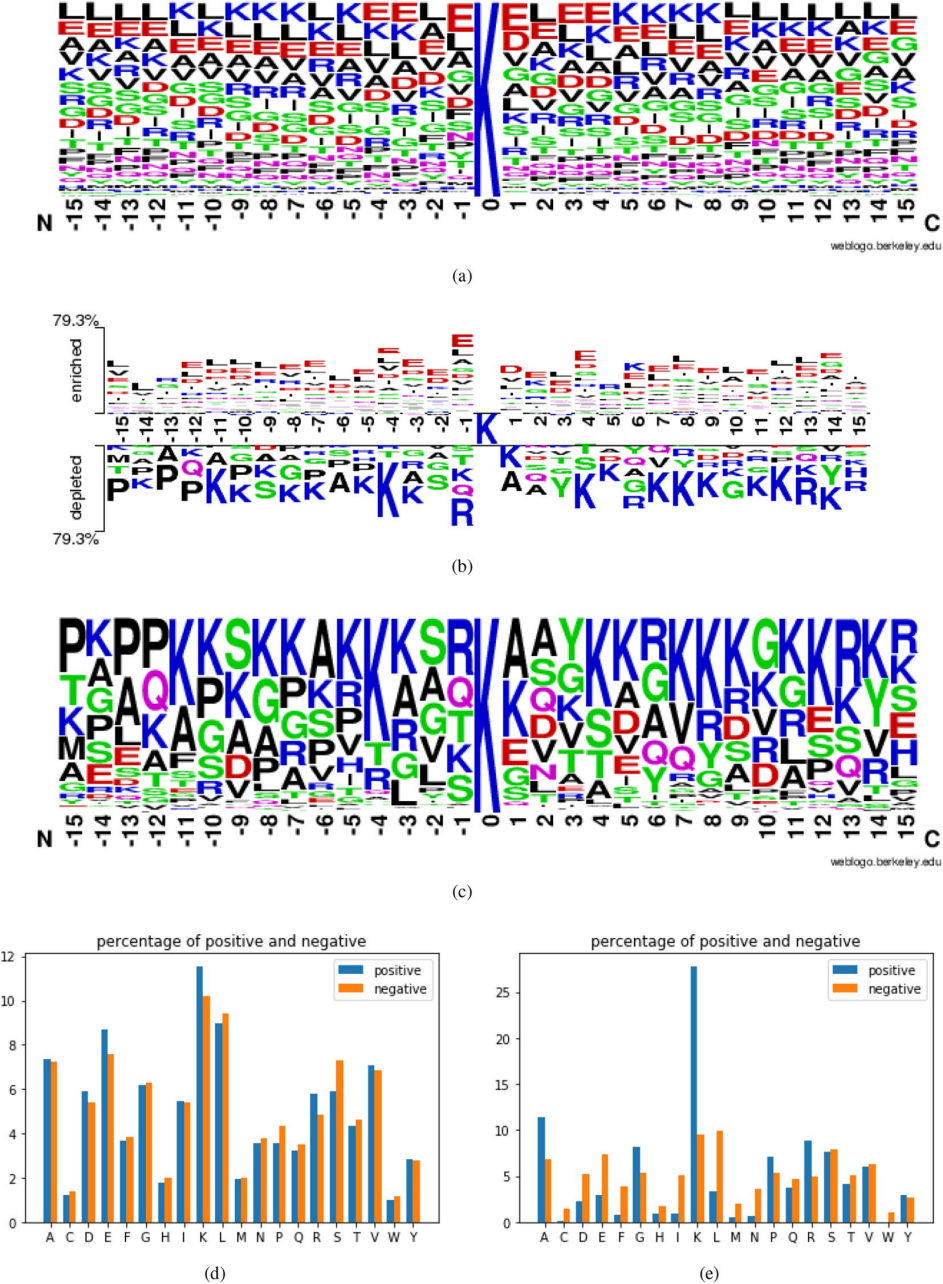
Position-specific amino acid composition analysis of crotonylated sequences and non-crotonylated sequences in plant dataset. (**a**) Indicates the Position-specific amino acid composition of crotonylated sequences in plant dataset based on the frequency plot of WebLogo. (**b**) Shows the Comparison of position-specific amino acid composition between crotonylated sequences in plant dataset (upper part) and crotonylated sequences in mammalian dataset (lower part) based on TwoSampleLogo. (**c**) Indicates the Position-specific amino acid composition of crotonylated sequences in mammalian dataset based on the frequency plot of WebLogo. (**d**) and (**e**) Shows the statistics of each amino acid composition (AAC) in plant and mammalian dataset respectively. From (**d**) it can be seen that large differences exist in the composition of K, E and S in plant dataset, and from (**e**) great differences exist among the composition of K, L, A, D, E and N in the dataset of mammalian.

The amino acid composition (AAC) was a widely used sequence-based feature for exploring the motif of residue components around the crotonylation sites^19,20^. Since comparing the AAC between positive and negative datasets, the residues containing significant differences could be regarded as useful attributes for crotonylation sites identification. The position-specific AAC neighbouring the crotonylation sites has been displayed by frequency plots of WebLogo^21^ in Fig. 1a–c. As illustrated in Fig. 1a, Lysine (K) and aspartic acid (E) are of significantly high abundance near the crotonylation sites in the plant sequences, while in the mammalian dataset, only K tends to occur more often near crotonylation sites as shown in Fig. 1c. Additionally, the differences among each AAC seem to be much larger in the mammalian dataset, which means that in the plant dataset, the differences among positive and negative samples with respect to AAC feature are not that obvious, that is the main reason that the performance in the mammalian dataset is much more outstanding than the plant dataset. The TwoSampleLogo graph was further illustrated to compare the differences of position-specific AAC between crotonylated sequences in the two datasets^22^. As shown in Fig. 1b, in the plant dataset, the most conserved motifs appeared to be associated with both positively charged residues, in particular K, and negatively charged composition, such as E. Additionally, the sequences of highly positively charged are involved in the residues of mammalian, typically K, which also occurred frequently in plant residues. Fig. 1d,e have indicated the occurrence of each amino acid composition in crotonylated and non-crotonylated sequences in the two datasets, and for crotonylation sites, the positively charged lysine (K) residue appeared to have the highest frequency around the substrate sites. Besides Fig. 1, the detailed figures of WebLogo and TwoSampleLogo of the plant dataset and the mammalian dataset are shown in Supplementary Figs. S1 and S2 in the supplementary materials.

### Performance on individual and incorporated features:

Based on the investigated features, SVM and RF classifiers were trained to determine the effectiveness of those features in identifying crotonylation sites. As shown in Table 1, the libsvm classifier trained with AAC reached an accuracy of 65% and an MCC value of 0.31, which is the lowest one. The AAPC feature performed slightly better than the AAC case, which achieved an accuracy of 66% and an MCC value of 0.33. For CKSAAP, the libsvm classifier yielded at a similar performance with AAPC but slightly higher in Sn. Among these features, the classifiers trained by EAAC and EGAAC features performed best for discriminating between crotonylated and non-crotonylated lysine residues, with EAAC classifier yielded a sensitivity, specificity, accuracy, and MCC value of 68%, 72%, 71% and 0.41, respectively and EGAAC yielded 74%, 66%, 70%, and 0.40 for the same criteria, respectively. Additionally, the ROC curve was generated to compare the predictive performance and stability of different classifiers in Supplementary Figs. S2 and S3 in supplementary materials.

**Table 1:** Performance on plant dataset.

Features	Dimension	Dataset	Classifier	Sn	Sp	Acc	MCC
AAC	20	Plant	libsvm	0.69	0.61	0.65	0.31
AAPC	400	Plant	libsvm	0.64	0.68	0.66	0.33
BE	620	Plant	libsvm	0.69	0.63	0.66	0.33
CKSAAP	1600	Plant	libsvm	0.65	0.67	0.66	0.32
EAAC	540	Plant	libsvm	0.68	0.72	0.71	0.40
EGAAC	135	Plant	libsvm	0.74	0.66	0.70	0.40
PSSM	620	Plant	libsvm	0.71	0.48	0.60	0.20
Incorporated$^{\text {a}}$	3935	Plant	libsvm	0.74	0.72	0.73	0.41
AAC	20	Plant	RF	0.68	0.60	0.64	0.28
AAPC	400	Plant	RF	0.58	0.69	0.64	0.28
BE	620	Plant	RF	0.73	0.63	0.68	0.36
CKSAAP	1600	Plant	RF	0.60	0.68	0.64	0.28
EAAC	540	Plant	RF	0.83	0.70	0.77	0.54
EGAAC	135	Plant	RF	0.82	0.69	0.75	0.51
PSSM	620	Plant	RF	0.70	0.60	0.65	0.31
Incorporated$^{\text {a}}$	3935	Plant	RF	0.85	0.73	0.79	0.57

$^{\text {a}}$Stands for the combination of each single feature, which means AAC + AAPC + BE + CKSAAP + EAAC + EGAAC + PSSM.

From the comparison among single features, the RF classifier trained from the EAAC feature gave the best performance, which gives the accuracy of 77%, MCC of 0.54 and AUC of 0.84 respectively. Feature EGAAC also achieved an accuracy of 74%, MCC 0.4 and AUC 0.78 in libsvm classifier, which is the best one among all features in libsvm method. Besides individual feature, incorporation of each feature was also carried out and achieved a good performance, with 71% accuracy, 0.40 MCC and 0.77 AUC of libsvm , and 77% accuracy and 0.55 MCC and 0.84 AUC of RF.

Moreover, the same procedure was also adopted for the mammalian dataset, and performance was shown in Table 2. Overall, the performance on the mammalian dataset was much more superior to on the plant dataset since the differences of amino acid between positive and negative are more obvious in the mammalian dataset, with the lowest accuracy on an individual feature at around 90%. Similar to the plant dataset, EAAC and EGAAC achieved the best performances among all features by giving the accuracy of 89% and around 90%, respectively. Due to its relatively small scale, the improvement of RF classifier over libsvm is not as outstanding as it has been when in plant dataset, but the overall performance of RF has yielded a high level, which not only gives an average ACC of each feature at around 90% but also AUC higher than 0.90. A comparison of this study and other existed tools were listed in Tables 3 and 4.

**Table 2:** Performance on mammalian dataset.

Features	Dimension	Dataset	Classifier	Sn	Sp	Acc	MCC
AAC	20	Mammalian	libsvm	0.90	0.87	0.88	0.76
AAPC	400	Mammalian	libsvm	0.98	0.76	0.87	0.75
BE	620	Mammalian	libsvm	0.83	0.93	0.88	0.76
CKSAAP	1600	Mammalian	libsvm	0.92	0.81	0.86	0.73
EAAC	540	Mammalian	libsvm	0.90	0.89	0.89	0.78
EGAAC	135	Mammalian	libsvm	0.90	0.93	0.91	0.83
PSSM	620	Mammalian	libsvm	0.98	0.91	0.94	0.85
Incorporated$^{\text {a}}$	3935	Mammalian	libsvm	1.0	0.85	0.92	0.86
AAC	20	Mammalian	RF	0.93	0.87	0.89	0.79
AAPC	400	Mammalian	RF	0.89	0.76	0.82	0.65
BE	620	Mammalian	RF	0.93	0.87	0.89	0.79
CKSAAP	1600	Mammalian	RF	0.93	0.81	0.86	0.73
EAAC	540	Mammalian	RF	0.93	0.87	0.89	0.79
EGAAC	135	Mammalian	RF	0.92	0.88	0.90	0.80
PSSM	620	Mammalian	RF	0.94	0.91	0.92	0.83
Incorporated$^{\text {a}}$	3935	Mammalian	RF	0.90	0.82	0.86	0.79

$^{\text {a}}$Stands for the combination of each single feature, which means AAC + AAPC + BE + CKSAAP + EAAC + EGAAC + PSSM.

**Table 3:** Performance comparison between our method and existing available crotonylation site prediction tools (pKcr).

Features	Dataset	Tool	Accuracy	Sensitivity	Specificity	MCC	AUC
AAC	Non-histone	pKcr	0.83	0.21	0.90	0.10	0.67
		This method	0.64	0.60	0.68	0.28	0.68
CKSAAP	Non-histone	pKcr	0.83	0.22	0.90	0.11	0.68
		This method	0.64	0.60	0.68	0.28	0.71
BE	Non-histone	pKcr	0.84	0.33	0.90	0.19	0.74
		This method	0.68	0.73	0.63	0.36	0.77
EAAC	Non-histone	pKcr	0.85	0.42	0.90	0.27	0.81
		This method	0.77	0.83	0.70	0.54	0.84
EGAAC	Non-histone	pKcr	0.85	0.42	0.90	0.25	0.81
		This method	0.77	0.83	0.70	0.51	0.82

The above comparison indicates that our study is more robust and gives a more balanced performance than the pKcr method.

**Table 4:** Performance comparison between our method and other two existing tools (CKSAAP_CrotSite and iKcr-PseEns).

Dataset	Method	Classifier	Feature	Sn	Sp	Acc	MCC	AUC
Mammalian	CKSAAP_CrotSite	libsvm	CKSAAP	0.92	0.99	0.98	0.92	0.99
	This paper	libsvm	CKSAAP	0.92	0.81	0.86	0.73	0.94
Mammalian	iKcr-PseEns	Ensemble Random Forest	PseAAC	0.90	0.95	0.94	0.81	0.97
	This paper	Random Forest	PseAAC	0.93	0.87	0.89	0.79	0.95

The above comparison indicated that our study works competitively good as these two published work.

### Performance on selected features

Feature selection based on Chi-square, LGBM and MRMD methods are involved in this study. For Chi-square method, We have selected the dimension of value whose p-value, which was transformed from Chi-square value, was less than 0.05 be kept for further training. A selection on the incorporated feature, which is originally of 3935 dimension, was also carried out, and those p-values less than 0.05 were kept, totally 100 dimension of features remained and the performance of selected incorporated features are listed in Table 5. Similar to the Chi-square method, the top-100 dimensions of feature in LGBM and MRMD methods were also selected. It can be seen that the selection over incorporated features has significant improvement in SVM method with accuracy rising from 71 to 74% and AUC from 0.77 to 0.81. For RF, there exists some improvement but not as large as shown in SVM, with accuracy improved from 74 to 77%, and AUC improved from 0.82 to 0.84. Moreover, in supplementary the performance on selected features of different classifiers has indicated in Supplementary Tables S4 and S5 for libsvm and RF classifiers respectively.

**Table 5:** Comparison of performance before and after feature selection method in the incorporated feature.

Selection method	Number of features	Classifier	Sn	Sp	Acc	MCC	AUC
Original	3935	svm	0.74	0.71	0.73	0.43	0.78
Chi-square	100		0.77	0.70	0.74	0.47	0.81
LGBM	100		0.77	0.75	0.76	0.45	0.83
MRMD	100		0.75	0.73	0.74	0.45	0.84
Original	3935	RF	0.83	0.65	0.74	0.49	0.82
Chi-square	100		0.84	0.70	0.77	0.55	0.84
LGBM	100		0.85	0.72	0.78	0.54	0.84
MRMD	100		0.83	0.70	0.76	0.55	0.83

Here ‘original’ corresponds to the incorporated feature, which is AAC + AAPC + BE + CKSAAP +EAAC + EGAAC + PSSM, of 3935 dimension. ‘Chi-square’ corresponds to the selected top-100 dimension of features after selection in Chi-square method, ‘LGBM’ corresponds to the selected top-100 dimension of features based on LGBM feature selection method, ‘MRMD’ corresponds to the selected top-100 dimension of features based on MRMD feature selection method.

**Figure 2:**
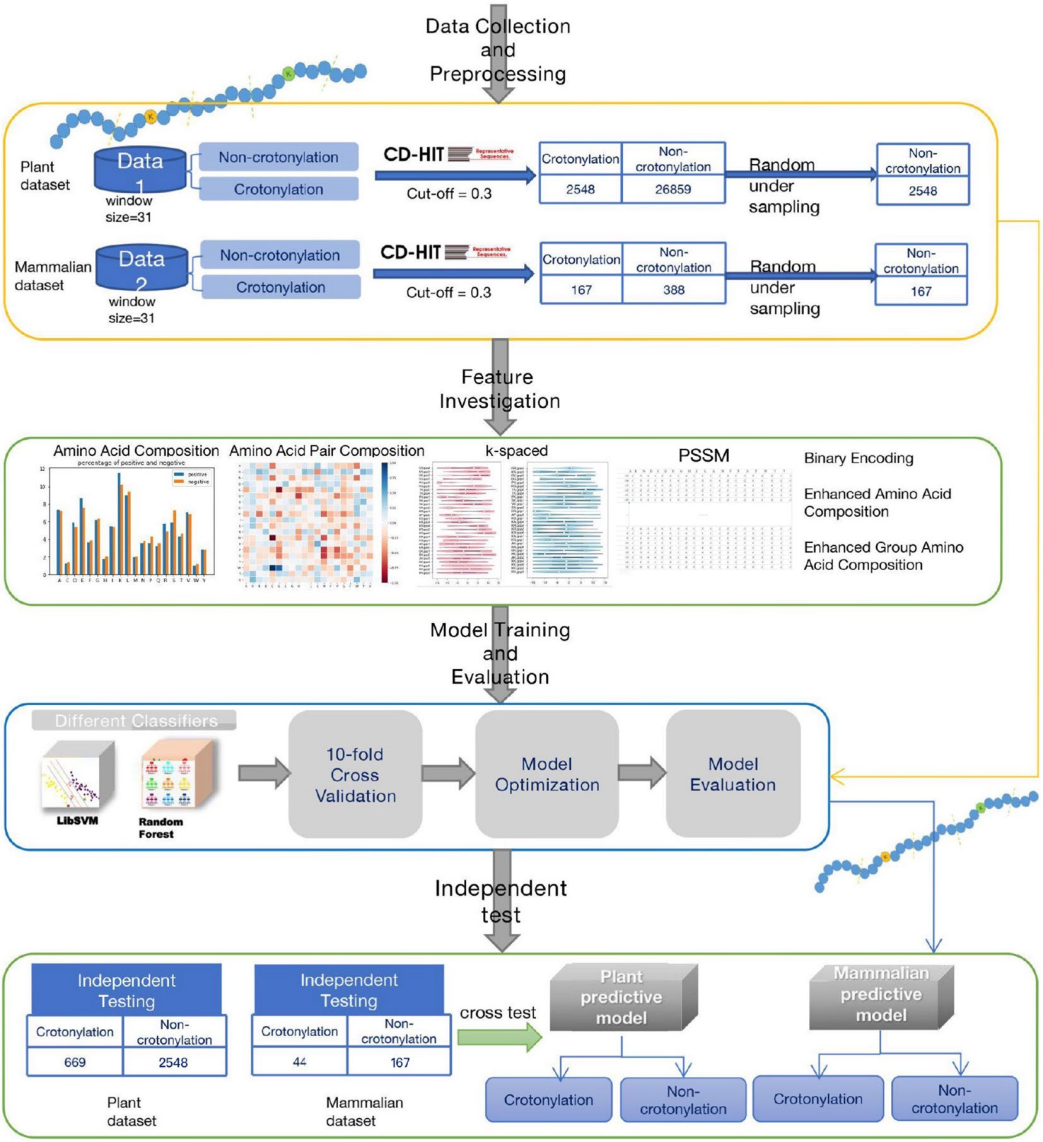
Flowchart of this paper. Four main steps contained: data collection and preprocessing, feature investigation, model training and evaluation and independent test.

Besides that, we have also carried a cross-species evaluation, which is to apply the trained classifier that obtained from the plant dataset onto the mammalian dataset, which can reflect that whether anything in common between these two species in crotonylation. The reason for using plant dataset as training set is due to its larger quantity of plant dataset. The steps in this cross-species evaluation are the same as previous training and testing procedures, but employed the whole mammalian dataset as the testing set this time. As shown in Table 6, the performance is not very promising, with most of the accuracy below the average rate of 50%. That is understandable as there are obviously differences that exist in the amino acid composition between species plant and mammalian(like what has been proposed in Fig. 2), which shows the species diversity.

**Table 6:** Performance of cross-species evaluation.

Training set	Feature	Validation set	Classifier	Sn	Sp	Acc	MCC	AUC
Plant	AAC	Mammalian	SVM	0.48	0.51	0.49	− 0.02	0.54
Plant	AAPC	Mammalian	SVM	0.11	0.57	0.34	− 0.36	0.30
Plant	BE	Mammalian	SVM	0.44	0.36	0.40	− 0.20	0.55
Plant	CKSAAP	Mammalian	SVM	0.26	0.54	0.40	− 0.20	0.39
Plant	EAAC	Mammalian	SVM	0.48	0.70	0.59	0.19	0.45
Plant	EGAAC	Mammalian	SVM	0.28	0.74	0.51	0.02	0.45
Plant	PSSM	Mammalian	SVM	0.15	0.55	0.35	− 0.33	0.35
Plant	AAC	Mammalian	RF	0.16	0.65	0.41	− 0.21	0.45
Plant	AAPC	Mammalian	RF	0.19	0.66	0.43	− 0.16	0.45
Plant	BE	Mammalian	RF	0.40	0.625	0.51	0.02	0.53
Plant	CKSAAP	Mammalian	RF	0.48	0.60	0.54	0.08	0.59
Plant	EAAC	Mammalian	RF	0.42	0.70	0.56	0.13	0.64
Plant	EGAAC	Mammalian	RF	0.33	0.71	0.52	0.05	0.63
Plant	PSSM	Mammalian	RF	0.21	0.68	0.45	− 0.14	0.47

In this evaluation, the plant dataset were treated as the training set and mammalian dataset as the testing set.

## Methods

A flowchart of this study was presented in Fig. 2, which contains four main steps: data collection and preprocessing, feature investigation, model training and evaluation and final independent test. The two datasets collected for the later process are listed in Supplementary Table S2 in supplementary materials, where the number of protein and sites in each dataset are shown. After obtaining these datasets, different types of sequence-based feature were extracted to encoding the sequences to multidimensional vectors for later training. Then, ten-fold cross-validation was utilized for evaluating the performances of predictors obtained from different machine learning methods. Finally, the classifier with the best predictive performance was further evaluated by an independent testing dataset. Details are described in the following sections.

### Data collection

In this study, one species of dataset used for the investigation of mammalian is from Universal Resource of Protein (UniProt) is for the investigation of the species mammalian, which contains 65 histone protein with 378 crotonylation sites^23^. Another dataset of the plant (*Carica papaya* L.) contains 5995 crotonylation sites located at 2120 non-histone protein sequences, which is available at http://www.bioinfogo.org/pkcr/download.php^17,18^.

To construct positive dataset for modeling, CD-HIT of threshold 30% were used for removing homologous protein sequences first as for high similarity of sequence may cause over-fitting in the training process. (2n + 1)-mer window size was segmented to extract fragmented sequences centered on the crotonylation sites with n neighbored amino acids upstream and downstream. The negative dataset was generated from non-crotonylation sites, on which those segmented sequences are centered on lysine residues without crotonylation annotation. 31-mer window size, where n = 15, was chosen after performing best for predicting of crotonylation sites based on the basic feature amino acid composition. Those sequences of length less than 31 amino acid compositions or those contain uncertain composition were filtered out, and a total of 3453 positives and 37,134 negatives segment sequences were obtained for plant dataset, and 379 positives and 500 negatives for mammalian dataset. Among each dataset, 80% and 20% were divided for training and testing dataset, respectively. Here 2548 positive and 26,859 negative sites in the training set of plant dataset, and 669 positives and 6720 negatives for the testing set. For the mammalian dataset, 167 positives and 388 negatives in the training set, and 44 positives and 95 negatives in the testing set. Both of these two datasets are very imbalanced in positive and negative, which would cause the performance of cross validation biased^24^, random under sampling method were employed in the training step, after which these two datasets contain equal-sized positive and negative sites in their training set, which means 2548 positive and 2548 negative samples in the plant dataset, 167 positive and 167 negative samples in the mammalian dataset.

### Feature extraction

In this study, sequence-based characterization of crotonylation were carried out. Sequence-schemed features were extracted, typically amino acid composition (AAC), amino acid pair composition (AAPC), binary encoding (BE), composition of k-spaced amino acid pair (CKSAAP), enhanced amino acid pair (EAAC), enhanced group amino acid pair (EGAAC) and position specific scoring matrix (PSSM)^25^.

#### AAC

AAC indicates the frequency of each amino acid occurs in a sequence. As there are 20 types of amino acid in a protein sequence, the dimension of an AAC feature is 20. For the sequences *x*, which is of fixed length *n* ($n=31$ in this study), the probability $P_{x}(k)$ of amino acid *k* is

$$\begin{aligned} P_{x}(k) = \frac{n_{x}(k)}{n}, \end{aligned}$$

where $n_{x}(k)$ refers to occurrence of amino acid *k*. The figure of position-specific amino acid in both the plant and the mammalian dataset has been indicated previously in Fig. 1d,e.

#### AAPC

Similar to AAC, AAPC shows the frequency of each amino acid pair occurs in the sequence. There are totally 20 types of amino acid in protein, hence $20 \times 20$ types of amino acid pairs available, so the dimension of AAPC feature should be 400. The probability $P_{x}(k)$ of amino acid pair in a sequence *x* is

$$\begin{aligned} P_{x}(k) = \frac{n_{x}(k)}{n*(n-1)}, \end{aligned}$$

where $n_{x}(k)$ is the occurrence of amino acid pair *k*. The figure of position-specific amino acid pairs in both the plant and the mammalian dataset has been indicated previously in Supplementary Fig. S6.

#### Binary encoding

Binary encoding is a straightforward way to encoding features, which is also known as “one-hot” encoding. Each amino acid corresponds to a vector of length 20 as there are possibly 20 types of amino acid in a protein sequence as mentioned. These 20 types of amino acid are sorted in a certain order, in this study alphabetic order, which is ‘ACDEFGHIKLMNPQRSTVWY’, was adopted as the target vector. For instance, ‘A’ will be reflected as a 20-dimensional vector ‘10000000000000000000’ (19 zeros here) and ‘C’ will be ‘01000000000000000000’(18 zeros after the digit ‘1’) etc. As an amino acid will be converted to a 20-dimensional vector, the output feature of binary encoding in this study should be 620 dimensional.

#### CKSAAP

CKSAAP is a criterion which is wildly used in the field of analysing of protein functions. When an integer *k* has been fixed, the number of k-spaced amino acid pair will be determined as containing the case that the gap between two neighbored amino acid ranges from 0 to the given integer *k*. CKSAAP indicates the frequency of amino acid pairs separated by any k composition. In this study k was chosen to be 4, which should contain the cases that k ranges from 0 to 4, meanwhile, as CKSAAP gives the same result as AAPC when k equals to 0, only cases in which k ranges from 1 to 4 are considered in this study, totally given 1600 dimension of feature for a single sequences. Supplementary Fig. S7a shows the general processing of CKSAAP generation, the comparison of CKSAAP in positive and negative samples of the plant and the mammalian dataset has been indicated in Supplementary Figs. S8 and S9 respectively in supplementary materials.

#### EAAC

EAAC was first raised by Chen in 2018^25^, where the AAC values are calculated based on the sequence window of fixed length (in this study, 5 was chosen) that continuously slides from the N- to C-terminus of each peptide. EAAC is calculated by

$$\begin{aligned} f(t,composition) = \frac{N(t, composition)}{N(composition)}, \end{aligned}$$

where

$$\begin{aligned}&t \subseteq \{A,C,D,...,Y\},\\&N(composition) \subseteq \{composition 1, composition 2, ... composition N\} \end{aligned}$$

in this study, there are 27 composition possible for each sequence, so totally the EAAC will give a 540 dimensional feature for each sequence. Supplementary Fig. S7b has shown the illustration of EAAC feature.

#### EGAAC

EGAAC is an enhanced feature of Group Amino Acid Composition, which was first raised by Lee in 2011^26^, where the 20 types of amino acid are further categorized into five classes according to their physicochemical properties, such as hydrophobicity, charge and molecular size(detailed list shown in Supplementary Table.S3). The calculation method of EGAAC is similar to EAAC, but focuses on the pair-wised amino acid in each group, which is:

$$\begin{aligned} f(p,composition) = \frac{N(p, composition)}{N(composition)}, \end{aligned}$$

where

$$\begin{aligned}&p \subseteq \{group1, group2, ..., group5\}, \\&composition \subseteq \{composition 1, composition 2, ... composition N\}. \end{aligned}$$

Here N(g, win) is the number of amino acids in group *g* within the sliding window *composition* and *N(composition)* is the size of sliding window. We have the number of composition equals to 27 as mentioned in EAAC part, hence each feature for EGAAC will give a 135 dimensional vector.

#### PSSM

PSSM is the short for Position Specific Scoring Matrix, which is a feature from the structural viewpoint, and has been extensively applied in the field protein secondary structure prediction^27^, subcellular localization^28^ and other bioinformatics analysis^29^.

As shown in Supplementary Fig.s8, PSSM profile of each sequence, which was generated by performing PSI-BLAST against the dataset of non-homologous crotonylated peptides, was composed of a matrix with $win*m$ elements, where *win* stands for the sequence length, *m* represents 20 types of amino acids. The PSSM profile matrix can be shown as:

$$\begin{aligned} Profile_{X} = \left[ \begin{matrix} P_{x,-15}(1)&{} \cdots &{}P_{x,+15}(m) \\ \vdots &{} \vdots &{} \ddots &{} \vdots \\ P_{x,+15}(1)&{} \cdots &{}P_{x,+15}(m) \\ \end{matrix} \right] . \end{aligned}$$

Then the $w*m$ matrix was transformed into a matrix with $20 \times 20$ features $S_{x}(i, j)$, where *i* and *j* range from 1 to 20, by summing up the rows that were involved in the same type of amino acid i. The feature matrix is indicated as:

$$\begin{aligned} PSSM_{x} = \left[ \begin{matrix} S_{x,-15}(1)&{} \cdots &{}S_{x,+15}(m) \\ \vdots &{} \vdots &{} \ddots &{} \vdots \\ S_{x,+15}(1)&{} \cdots &{}S_{x,+15}(m) \\ \end{matrix} \right] . \end{aligned}$$

Finally, each element in the feature matrix $PSSM_{x}$ was normalized using a Sigmoid function^30^, and $\Phi (x)$ can be written as:

$$\begin{aligned} \Phi _{x}(i, j) = \frac{1}{1+\exp {\frac{-S_{x}(i, j)}{w}}} \end{aligned}$$

$w = 31$ amino acid of each sequence in this study, so the sequence length ranges from $-15$ to $+15$ in this study.

### Feature selection

For the aim of improving prediction performance and removing redundant features for speeding up the prediction process, feature selection is a phase which is of paramount importance. In the feature selection procedure, each dimension of the feature vectors was ranked according to certain criterion of “importance”, then those are of lower “importance” would be deleted, then the feature vector will be of lower dimension but higher importance, which is more information-rich than the original encoding feature. In this study, the Chi-square value method and the light gradient boosting machine(LGBM) feature selection method are listed.

In the ranking step of the Chi-square value method in this study, Chi-square value of each feature was calculated, then according to the Chi-square value, p-value for each dimension of the individual feature was obtained and whose p-value greater than 0.05 was removed. This selection was taken on each feature for deleting those redundant dimensions in each type of feature. 15 out of 20 dimensions of AAC, 80 out of 400 from AAPC, 55 out of BE, 80 out of 1600 from CKSAAP, 100 out of 540 from EAAC, 93 out of 135 from EGAAC and 101 out of 620 from PSSM were selected.

LGBM is a highly efficient gradient boosting decision tree, suitable for scenarios with large amounts of data and high-dimensional features^31^. The embedded approach is similar to the wrapper approach but seeks the optimal features subset by a built-in classification algorithm^32^. In this work, the LGBM wrapper^31^ was used for feature selection. Its purpose was to feed the LGBM model with training data and to determine and rank the feature importance values, in order to select those features with importance values greater than the average. This step used the python toolkit from https://lightgbm.readthedocs.io^33^. Based on LGBM method, 10 out of 20 dimensions of AAC, 70 out of 400 from AAPC, 65 out of BE, 85 out of 1600 from CKSAAP, 109 out of 540 from EAAC, 87 out of 135 from EGAAC and 120 out of 620 from PSSM were selected.

For both Chi-square method and LGBM method are mainly focused on the improvement of the classification accuracy, the stability of dimension reduction may be ignored, then we have enrolled Max-Relevance-Max-Distance (MRMD) feature ranking method^34^, which balances accuracy and stability of feature ranking and prediction task. For this method, it computes the maximum-relevance-maximum-distance of each dimension. A Java-based package from http://lab.malab.cn/soft/MRMD/contact.html can be found for the ranking process^34,35^. In MRMD method, the selection model type can be selected among three options: rf, SVM and bagging, in this study rf was chosen.Another parameter, which is the distance function used in this method of calculation, could be selected among 1 for Euclidean distance, 2 for Cosine distance, 3 for Tanimoto distance and 4 for mean. In this study, 1 for Euclidean distance was chosen. Based on MRMD method, different dimensions of selected features were tried, among which the best-performed cases in every attribute were kept. In that case, 16 out of 20 dimensions of AAC, 73 out of 400 from AAPC, 45 out of BE, 220 out of 1600 from CKSAAP, 150 out of 540 from EAAC, 100 out of 135 from EGAAC and 150 out of 620 from PSSM were selected.

For the reason that some features might of higher importance than others, for instance, some dimensions in EGAAC might of higher Chi-square values than some dimensions in AAC. Considering that, the selection of the total incorporated feature has been carried out. The incorporated feature is of dimension 20 (AAC) + 620 (BE) + 1600 (CKSAAP) + 540 (EAAC) + 135 (EGAAC) = 3935, and then those of top-100 Chi-square value dimensions were kept afterwards as these 100 dimensions are of p-value less than 0.05. Similar to the Chi-square method, top-100 features from LGBM and MRMD method are also selected. The statistics of selection of incorporated feature are shown on Supplementary Fig. S3–S4.

After feature selection we have efficiently reduced the dimension of features and improvement in performance. In Supplementary Table S1–S6 of supplementary materials, performances of the three feature selection methods are attached.

### Model construction

This study involves machine learning method is the prediction of crotonylation. Support vector machine (SVM) and Random Forest (RF) methods are adopted.

As a classical machine learning method, SVM is the most-often-used method for classification problems which are of enough data but not as plenty as required for deep learning method. It is a supervised learning method which was first proposed in 1963 by Vapnik and Lerner in the field of pattern recognition^36^. After developed in decades, it is still the top-used machine learning method in binary-class-division. SVM is based on associated learning algorithms using regression analysis to classify data^37^, the main idea is to find a boundary which can separate samples into different parts.

In this study, the SVM with radial basis function (RBF) kernel was adopted. Penalty parameter *C* was selected from set $\{2^{0}, 2^{1},2^{2},\dots , 2^{10}\}$ and the kernel parameter $\gamma$ was selected from set $\{2^{-10}, 2^{-9},2^{-8},\dots , 2^{0}\}$ by grid searching. The SVM classifier was developed by using the python module ‘sklearn’^38^.

RF method is another wildly-adopted method in the field of machine learning, which was first proposed in 2001 by Breiman, L^39^. It is a combination of tree predictors such that each tree depends on the values of a random vector sampled independently and with the same distribution for all trees in the forest. RF is more advanced than traditional machine learning method as it can work efficiently in more complicated cases and gives out a more balanced result when imbalanced dataset provided. The training process of RF was by setting the tree number from set $\{1400, 1600, 1800, ..., 2400\}$, and it is also implemented based on python module ‘sklearn’^38^.

### Performance evaluation

In the generation of machine learning classifier, the k-fold cross-validation was employed to evaluate their predictive performances. When implementing k-fold cross-validation, all the training data, including positive and negative sequences, were randomly clustered into k equal-sized subgroups. After that k-1 of them shall be regarded as the training sample and the remaining one subgroup was considered as the validation sample. In a round of k-fold cross-validation, each of the k subgroups should be considered as the validation sample once in turn. In this study, k equals 10 was chosen for the cross validation.

Sensitivity (Sn), specificity (Sp), accuracy (Acc), and Matthews correlation coefficient (MCC) have been used as the metrics to determine the performance of the generated models. The four metrics are defined in terms of where TP, FN, TN, and FP denote the instances of true positive, false negative, true negative, and false positives, respectively as:

$$\begin{aligned} Sn= & {} \frac{TP}{TP+FN}\\ Sp= & {} \frac{TN}{TN+FP}\\ Acc= & {} \frac{TP+TN}{TP+FP+TN+FN}\\ MCC= & {} \frac{(TP \times TN)-(FN \times FP)}{\sqrt{(TP+FN) \times (TN+FP) \times (TP+FP) \times (TN+FN)}}. \end{aligned}$$

ROC curve is also adopted as an evaluation criterion in this study as a more objective measurement than sensitivity and specificity. The area under curve (AUC) is an important criterion in performance evaluation for imbalanced cases. After evaluating the of k-fold cross-validation, the classifier which achieved the best predictive performance was further evaluated by an independent testing dataset that was not included at all in the training samples.

### Independent test

For generalization evaluation and performance comparison with the baseline method, an independent test is necessary to further evaluate of the performance^40^. In this study, the testing set was 20% non-overlapped part from the whole dataset, which contains 669 crotonylated and 2548 non-crotonylated samples from the plant dataset, 44 positive and 167 negative from the mammalian set. Moreover, a comparison between the existed method and this study in terms of predictive performance was also performed. Besides this, a cross-species validation has been involved, in which the classifier obtained from plant dataset was used for classification of mammalian samples, to see whether the classifier for the sample types of PTM which obtained from one species would work in another.

## Conclusion

Since the release of experiment-verified crotonylation sites in different species has provided more samples in crotonylation database, we have carried out a set of experiments for predicting of crotonylated and non-crotonylation sites by using machine learning method, aiming to give a more balanced performance in different species of datasets. The methods of classifiers SVM and RF have achieved competitively good performances as existed methods in both plant and mammalian datasets, which has filled the gap with no related research on different species. In this study, SVM tends to be more efficient in the mammalian dataset as the quantity of mammalian samples is relatively small. RF classifier could work much more efficiently than SVM in the plant dataset with various kinds of features, especially EGAAC, which has shown great accuracy and robustness in the classification task, with accuracy 70%, 90% and AUC 0.84, 0.98 in plant and mammalian dataset respectively. Feature selection provided slightly improved and more robust result than the previously proposed method. Besides, a cross-species classification task was also involved in this study, to see whether the classifier trained from one species of these two employed datasets could work well in the other, which proves the diversity of different species. But with the limitation of sample data, on the one hand, the differences between positive and negative samples in the perception of position-specific AAC is not quite large in the plant dataset, which makes the performance of the plant dataset much lower than on the mammalian dataset. On the other hand, the number of samples of these two datasets, is large enough for traditional machine learning method, but not as many as required for more advanced study such as deep learning, and that is a reason why those classification methods employed in this study are relatively traditional and not that up-to-date methods. However, these drawbacks can be chased up when more experiment-verified data released. As if more verified data were released, some advanced deep learning methods with neural networks can be employed for further study, also, more features that can reflect or even enhance the differences between positive and negative would be used, which can make up the relatively low performance caused by the sample components.

## Supplementary information

Supplementary Information.

## References

[1] Mann M, Jensen ON (2003). Proteomic analysis of post-translational modifications. Nat. Biotechnol..

[2] Huang H (2017). iPTMnet: An integrated resource for protein post-translational modification network discovery. Nucleic Acids Res..

[3] Gong F, Miller KM (2013). Mammalian DNA repair: Hats and HDACS make their mark through histone acetylation. Mutat. Res. Fund. Mol. Mech. Mutagen..

[4] Filtz TM, Vogel WK, Leid M (2014). Regulation of transcription factor activity by interconnected post-translational modifications. Trends Pharmacol. Sci..

[5] Hornbeck PV (2015). Phosphositeplus, 2014: Mutations, PTMS and recalibrations. Nucleic Acids Res..

[6] Li X (2012). Quantitative chemical proteomics approach to identify post-translational modification-mediated protein-protein interactions. J. Am. Chem. Soc..

[7] Vermeulen M, Hubner NC, Mann M (2008). High confidence determination of specific protein-protein interactions using quantitative mass spectrometry. Curr. Opin. Biotechnol..

[8] Zamaraev AV, Kopeina GS, Prokhorova EA, Zhivotovsky B, Lavrik IN (2017). Post-translational modification of caspases: The other side of apoptosis regulation. Trends Cell Biol..

[9] Urdinguio RG (2019). Chromatin regulation by histone h4 acetylation at lysine 16 during cell death and differentiation in the myeloid compartment. Nucleic Acids Res..

[10] Cruz ER, Nguyen H, Nguyen T, Wallace IS (2019). Functional analysis tools for post-translational modification: A post-translational modification database for analysis of proteins and metabolic pathways. Plant J..

[11] Romero-Puertas, M. C. & Sandalio, L. M. Role of no-dependent posttranslational modifications in switching metabolic pathways. In *Advances in Botanical Research*, vol. 77, 123–144 (Elsevier, Amsterdam, 2016).

[12] Huang K-Y (2016). 10-year anniversary of a resource for post-translational modification of proteins. Nucleic Acids Res..

[13] Tan M (2011). Identification of 67 histone marks and histone lysine crotonylation as a new type of histone modification. Cell.

[14] Huang K-Y (2019). exploring disease association and cross-talk of post-translational modifications. Nucleic Acids Res..

[15] Ju Z, He JJ (2017). Prediction of lysine crotonylation sites by incorporating the composition of k-spaced amino acid pairs into chou’s general pseaac. J. Mol. Graph. Model..

[16] Qiu W-R (2018). ikcr-pseens: Identify lysine crotonylation sites in histone proteins with pseudo components and ensemble classifier. Genomics.

[17] Liu K (2018). A qualitative proteome-wide lysine crotonylation profiling of (Carica papaya L.). Sci. Rep..

[18] Zhao Y, He N, Chen Z, Li L (2020). Identification of protein lysine crotonylation sites by a deep learning framework with convolutional neural networks. IEEE Access.

[19] Kao H-J, Nguyen V-N, Huang K-Y, Chang W-C, Lee T-Y (2020). Succsite: Incorporating amino acid composition and informative k-spaced amino acid pairs to identify protein succinylation sites. Genom. Proteom. Bioinform..

[20] Huang K-Y, Kao H-J, Hsu JB-K, Weng S-L, Lee T-Y (2019). Characterization and identification of lysine glutarylation based on intrinsic interdependence between positions in the substrate sites. BMC Bioinform..

[21] Crooks GE, Hon G, Chandonia J-M, Brenner SE (2004). Weblogo: A sequence logo generator. Genome Res..

[22] Vacic V, Iakoucheva LM, Radivojac P (2006). Two Sample Logo: A graphical representation of the differences between two sets of sequence alignments. Bioinformatics.

[23] Malebary SJ, Rehman MSU, Khan YD (2019). icrotok-pseaac: Identify lysine crotonylation sites by blending position relative statistical features according to the chou’s 5-step rule. PLoS One.

[24] He H, Garcia EA (2009). Learning from imbalanced data. IEEE Trans. Knowl. Data Eng..

[25] Chen Z (2018). iFeature: A Python package and web server for features extraction and selection from protein and peptide sequences. Bioinformatics.

[26] Lee T-Y, Lin Z-Q, Hsieh S-J, Bretaña NA, Lu C-T (2011). Exploiting maximal dependence decomposition to identify conserved motifs from a group of aligned signal sequences. Bioinformatics.

[27] Lee T-Y, Chen S-A, Hung H-Y, Ou Y-Y (2011). Incorporating distant sequence features and radial basis function networks to identify ubiquitin conjugation sites. PLoS One.

[28] Hsu JB-K, Bretaña NA, Lee T-Y, Huang H-D (2011). Incorporating evolutionary information and functional domains for identifying RNA splicing factors in humans. PLoS One.

[29] Xie D, Li A, Wang M, Fan Z, Feng H (2005). LOCSVMPSI: A web server for subcellular localization of eukaryotic proteins using SVM and profile of PSI-BLAST. Nucleic Acids Res..

[30] Huang K-Y, Hsu JB-K, Lee T-Y (2019). Characterization and identification of lysine succinylation sites based on deep learning method. Sci. Rep..

[31] Ke, G. *et al.* Lightgbm: A highly efficient gradient boosting decision tree. *Advances in Neural Information Processing Systems* 3146–3154 (2017).

[32] Lee H (2019). Stage-specific requirement for mettl3-dependent m6a mRNA methylation during haematopoietic stem cell differentiation. Nat. Cell Biol..

[33] Lv Z, Wang D, Ding H, Zhong B, Xu L (2020). Escherichia coli DNA n-4-methycytosine site prediction accuracy improved by light gradient boosting machine feature selection technology. IEEE Access.

[34] Zou Q, Zeng J, Cao L, Ji R (2016). A novel features ranking metric with application to scalable visual and bioinformatics data classification. Neurocomputing.

[35] Zou Q, Wan S, Ju Y, Tang J, Zeng X (2016). Pretata: Predicting tata binding proteins with novel features and dimensionality reduction strategy. BMC Syst. Biol..

[36] Vapnik V, Lerner AY (1963). Recognition of patterns with help of generalized portraits. Avtomat. i Telemekh.

[37] Vapnik VN (1999). An overview of statistical learning theory. IEEE Trans. Neural Netw..

[38] Chen Z (2020). ilearn: An integrated platform and meta-learner for feature engineering, machine-learning analysis and modeling of dna, rna and protein sequence data. Brief. Bioinform..

[39] Breiman L (2001). Random forests. Mach. Learn..

[40] Wang H, Wang Z, Li Z, Lee T-Y (2020). Incorporating deep learning with word embedding to identify plant ubiquitylation sites. Front. Cell Dev. Biol..

