# Supplementary material for: Characterization and identification of lysine crotonylation sites based on machine learning method on both plant and mammalian
Source: Sci Rep. 2020 Nov 24;10:20447. doi: 10.1038/s41598-020-77173-0 (PMC7686339; doi:10.1038/s41598-020-77173-0)
Supplement: Supplementary file 1 — Supplementary Information. [file 41598_2020_77173_MOESM1_ESM.pdf]

Characterization and Identification of Lysine Crotonylation Sites based on Machine Learning Method on both Plant and Mammalian.

Authors: Rulan Wang†, Zhuo Wang†, Hongfei Wang, Yuxuan Pang, and Tzong-Yi Lee*

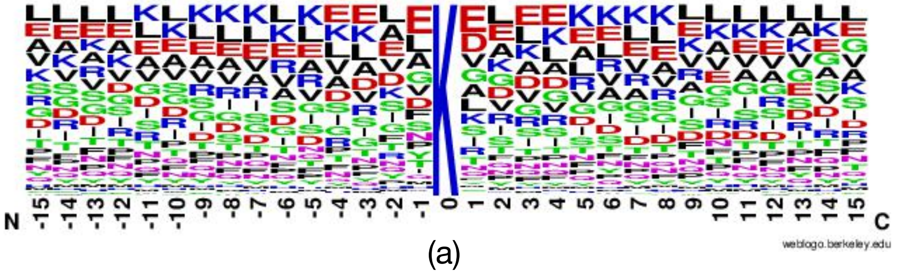

(a)

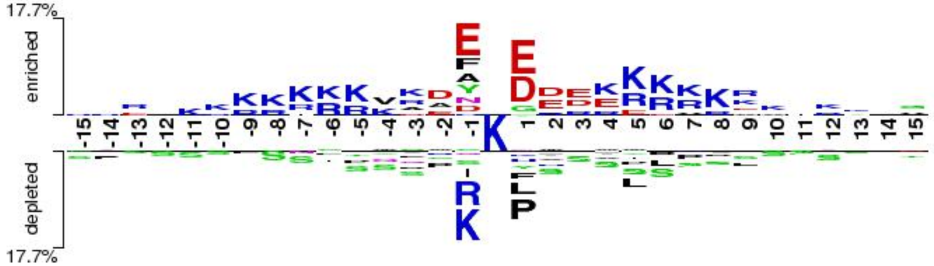

(b)

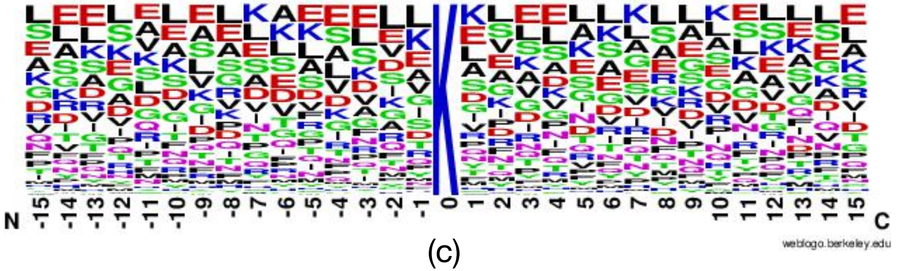

(c)

Figure s1: Position-specific amino acid composition of crotonylated sites in sequence of plant dataset. (a) : Position-specific amino acid composition of crotonylated sequences based on the frequency plot of WebLogo. (B) Comparison of position-specific amino acid composition between crotonylated and non-crotonylated sequences based on TwoSampleLogo analysis. (C) Position-specific amino acid composition of non-crotonylated sequences based on the frequency plot of WebLogo.

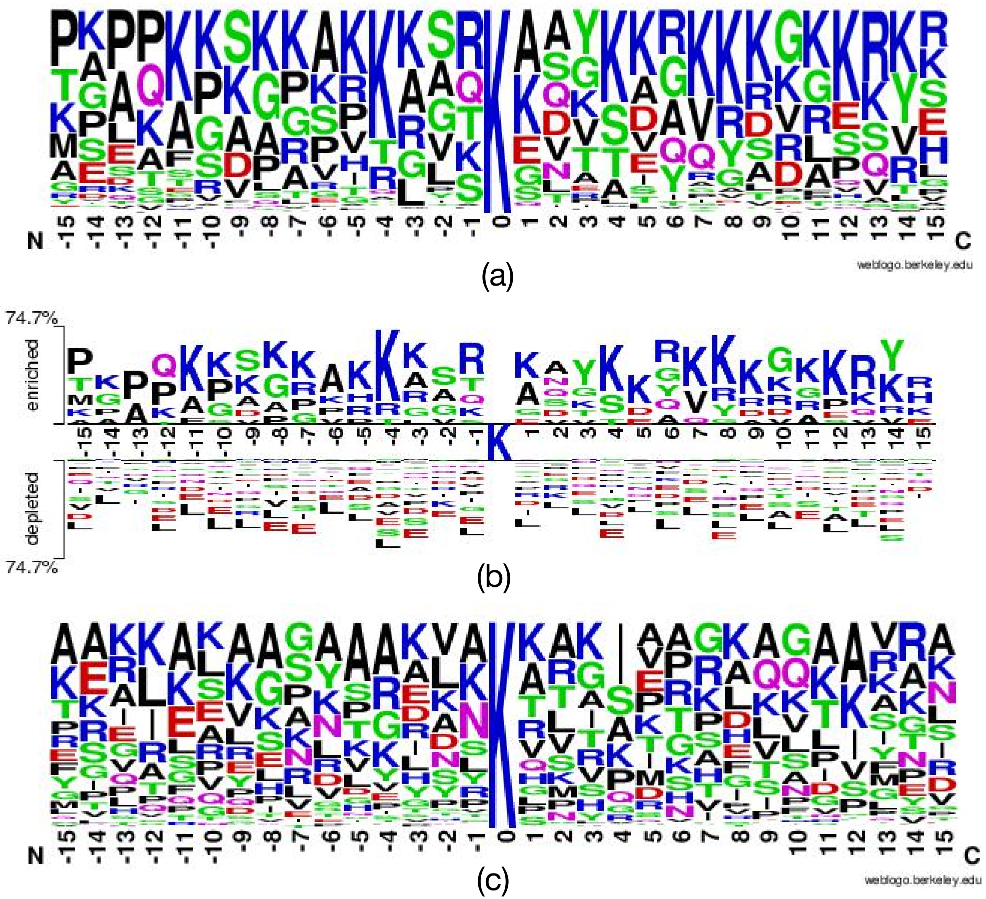

Figure s2: Position-specific amino acid composition of crotonylated sites in sequence of mammalian dataset. (a) : Position-specific amino acid composition of crotonylated sequences based on the frequency plot of WebLogo. (B) Comparison of position-specific amino acid composition between crotonylated and non-crotonylated sequences based on TwoSampleLogo analysis. (C) Position-specific amino acid composition of non-crotonylated sequences based on the frequency plot of WebLogo.

		Dimension	Dataset	Classifier	Sn	Sp	Acc	MCC	AUC
AAC	Original	20	Plant	libsvm	0.69	0.61	0.65	0.31	0.70
	Chi-square	15	Plant	libsvm	0.72	0.58	0.65	0.30	0.70
AAPC	Original	400	Plant	libsvm	0.64	0.68	0.66	0.33	0.72
	Chi-square	80	Plant	libsvm	0.65	0.68	0.66	0.33	0.72
BE	Original	620	Plant	libsvm	0.69	0.63	0.66	0.33	0.73
	Chi-square	55	Plant	libsvm	0.68	0.68	0.68	0.36	0.75
CKSAAP	Original	1600	Plant	libsvm	0.65	0.67	0.66	0.32	0.73
	Chi-square	80	Plant	libsvm	0.66	0.68	0.67	0.33	0.73
EAAC	Original	540	Plant	libsvm	0.68	0.72	0.71	0.40	0.78
	Chi-square	100	Plant	libsvm	0.69	0.71	0.70	0.39	0.78
EGAAC	Original	135	Plant	libsvm	0.74	0.66	0.70	0.40	0.76
	Chi-square	93	Plant	libsvm	0.75	0.66	0.70	0.41	0.78
PSSM	Original	620	Plant	libsvm	0.71	0.48	0.60	0.20	0.64
	Chi-square	101	Plant	libsvm	0.75	0.67	0.71	0.41	0.76
AAC+AAPC+BE+CKSAAP+EAAC+EGAAC+PSSM	Original	3935	Plant	libsvm	0.74	0.71	0.73	0.43	0.78
	Chi-square	524	Plant	libsvm	0.75	0.70	0.73	0.43	0.79

Table s1: Comparison of performance on the original feature and Chi-square method selected feature with libsvm classifier. Here column “Dimension” shows the dimension of corresponding feature vectors before and after feature selection of each attribute. Slightly improvement can be seen in each attribute.

		Dimension	Dataset	Classifier	Sn	Sp	Acc	MCC	AUC
AAC	Original	20	Plant	libsvm	0.69	0.61	0.65	0.31	0.70
	Chi-square	15	Plant	libsvm	0.72	0.58	0.65	0.30	0.70
AAPC	Original	400	Plant	libsvm	0.64	0.68	0.66	0.33	0.72
	Chi-square	80	Plant	libsvm	0.65	0.68	0.66	0.33	0.72
BE	Original	620	Plant	libsvm	0.69	0.63	0.66	0.33	0.73
	Chi-square	55	Plant	libsvm	0.68	0.68	0.68	0.36	0.75
CKSAAP	Original	1600	Plant	libsvm	0.65	0.67	0.66	0.32	0.73
	Chi-square	80	Plant	libsvm	0.66	0.68	0.67	0.33	0.73
EAAC	Original	540	Plant	libsvm	0.68	0.72	0.71	0.40	0.78
	Chi-square	100	Plant	libsvm	0.69	0.71	0.70	0.39	0.78
EGAAC	Original	135	Plant	libsvm	0.74	0.66	0.70	0.40	0.76
	Chi-square	93	Plant	libsvm	0.75	0.66	0.70	0.41	0.78
PSSM	Original	620	Plant	libsvm	0.71	0.48	0.60	0.20	0.72
	Chi-square	101	Plant	libsvm	0.75	0.67	0.71	0.41	0.76
AAC+AAPC+BE+CKSAAP +EAAC+EGAAC+PSSM	Original	3935	Plant	libsvm	0.74	0.71	0.73	0.43	0.78
	Chi-square	524	Plant	libsvm	0.75	0.70	0.73	0.43	0.79

Table s2: Comparison of performance on the original feature and Chi-square method selected feature with RF classifier. Here column “Dimension” shows the dimension of corresponding feature vectors before and after feature selection of each attribute. It can be seen that the improvement before and after the feature selection process is not as obvious as in the case of SVM classifier.

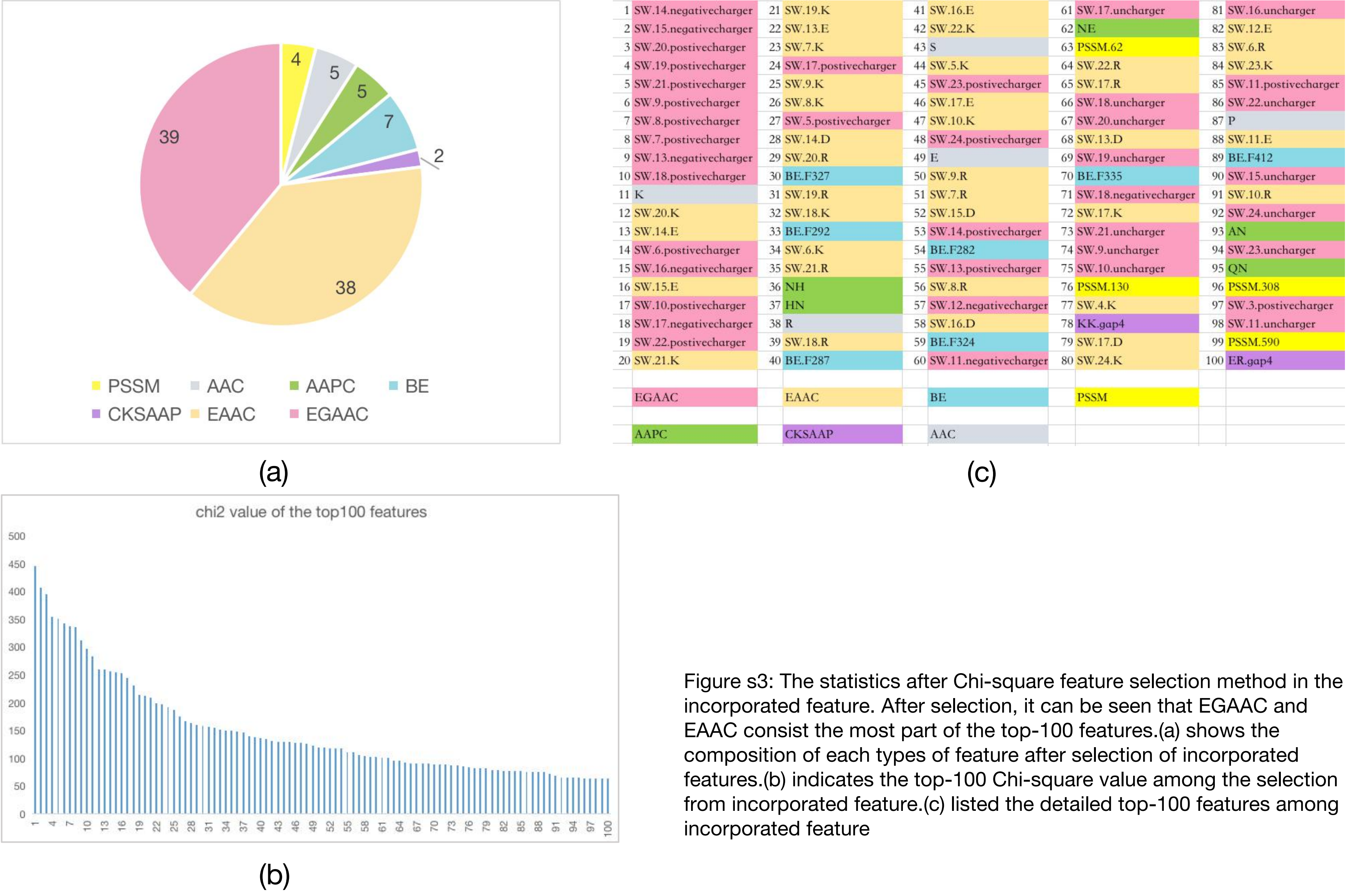

Figure s3: The statistics after Chi-square feature selection method in the incorporated feature. After selection, it can be seen that EGAAC and EAAC consist the most part of the top-100 features.(a) shows the composition of each types of feature after selection of incorporated features.(b) indicates the top-100 Chi-square value among the selection from incorporated feature.(c) listed the detailed top-100 features among incorporated feature

		Dimension	Dataset	Classifier	Sn	Sp	Acc	MCC	AUC
AAC	Original	20	Plant	libsvm	0.69	0.61	0.65	0.31	0.70
	LGBM	15	Plant	libsvm	0.72	0.65	0.69	0.30	0.72
AAPC	Original	400	Plant	libsvm	0.64	0.68	0.66	0.33	0.72
	LGBM	80	Plant	libsvm	0.67	0.71	0.69	0.33	0.73
BE	Original	620	Plant	libsvm	0.69	0.63	0.66	0.33	0.73
	LGBM	55	Plant	libsvm	0.66	0.70	0.68	0.37	0.75
CKSAAP	Original	1600	Plant	libsvm	0.65	0.67	0.66	0.32	0.73
	LGBM	80	Plant	libsvm	0.68	0.68	0.66	0.33	0.75
EAAC	Original	540	Plant	libsvm	0.68	0.72	0.71	0.40	0.78
	LGBM	100	Plant	libsvm	0.71	0.71	0.71	0.40	0.78
EGAAC	Original	135	Plant	libsvm	0.74	0.66	0.70	0.40	0.76
	LGBM	93	Plant	libsvm	0.76	0.65	0.71	0.41	0.78
PSSM	Original	620	Plant	libsvm	0.71	0.48	0.60	0.20	0.64
	LGBM	101	Plant	libsvm	0.78	0.69	0.73	0.41	0.77
AAC+AAPC+BE+CKSAAP+EAAC+EGAAC+PSSM	Original	3935	Plant	libsvm	0.74	0.71	0.73	0.43	0.78
	LGBM	524	Plant	libsvm	0.76	0.75	0.75	0.43	0.80

Table s3: Comparison of performance on the original feature and LGBM method selected feature with libsvm classifier. Here column “Dimension” shows the dimension of corresponding feature vectors before and after feature selection of each attribute. Slightly improvement can be seen in each attribute.

		Dimension	Dataset	Classifier	Sn	Sp	Acc	MCC	AUC
AAC	Original	20	Plant	libsvm	0.69	0.61	0.65	0.31	0.70
	LGBM	15	Plant	libsvm	0.73	0.59	0.66	0.30	0.70
AAPC	Original	400	Plant	libsvm	0.64	0.68	0.66	0.33	0.72
	LGBM	80	Plant	libsvm	0.64	0.70	0.67	0.33	0.72
BE	Original	620	Plant	libsvm	0.69	0.63	0.66	0.33	0.73
	LGBM	55	Plant	libsvm	0.69	0.70	0.69	0.36	0.76
CKSAAP	Original	1600	Plant	libsvm	0.65	0.67	0.66	0.32	0.73
	LGBM	80	Plant	libsvm	0.67	0.68	0.67	0.33	0.74
EAAC	Original	540	Plant	libsvm	0.68	0.72	0.71	0.40	0.78
	LGBM	100	Plant	libsvm	0.70	0.72	0.71	0.39	0.79
EGAAC	Original	135	Plant	libsvm	0.74	0.66	0.70	0.40	0.76
	LGBM	93	Plant	libsvm	0.77	0.69	0.72	0.41	0.78
PSSM	Original	620	Plant	libsvm	0.71	0.48	0.60	0.20	0.72
	LGBM	101	Plant	libsvm	0.77	0.70	0.73	0.41	0.77
AAC+AAPC+BE+CKSAAP +EAAC+EGAAC+PSSM	Original	3935	Plant	libsvm	0.74	0.71	0.73	0.43	0.78
	LGBM	524	Plant	libsvm	0.76	0.71	0.75	0.43	0.79

Table s4: Comparison of performance on the original feature and LGBM method selected feature with RF classifier. Here column “Dimension” shows the dimension of corresponding feature vectors before and after feature selection of each attribute. It can be seen that the improvement before and after the feature selection process is not as obvious as in the case of SVM classifier.

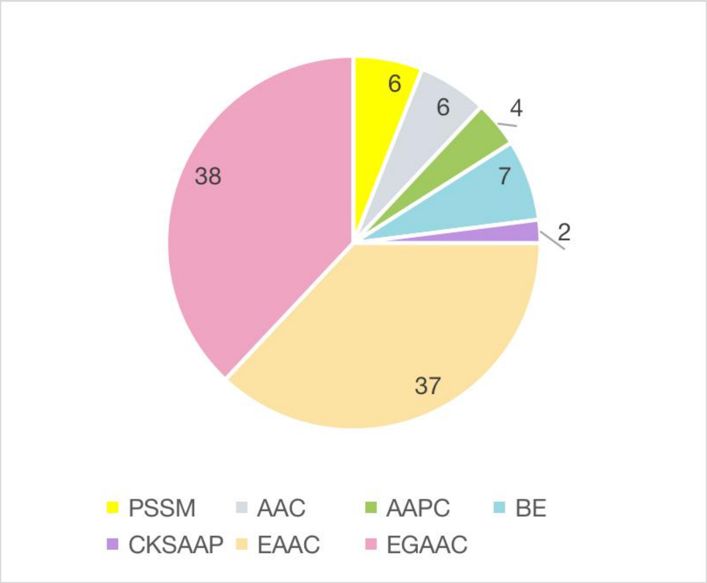

(a)

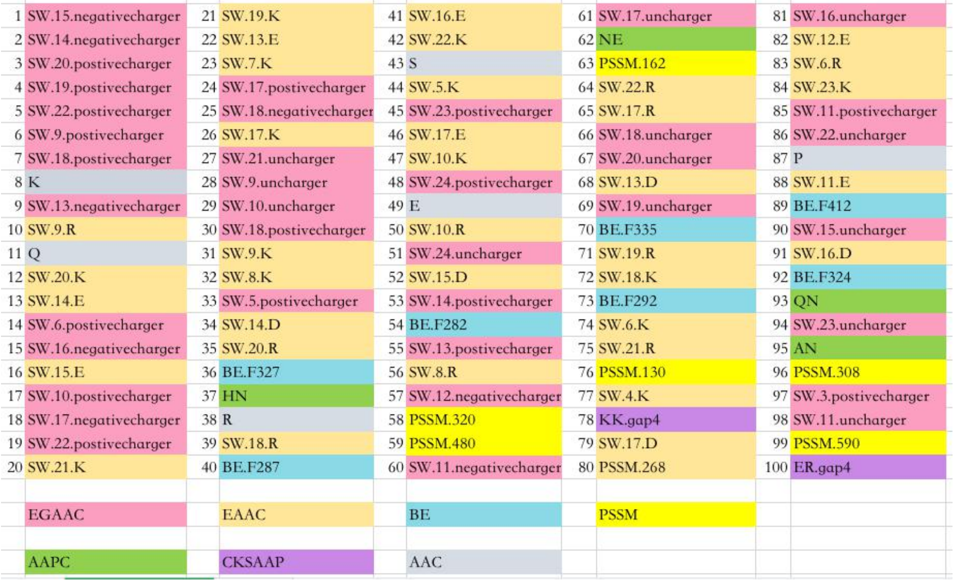

1	SW.15.negativecharger	21	SW.19.K	41	SW.16.E	61	SW.17.uncharger	81	SW.16.uncharger
2	SW.14.negativecharger	22	SW.13.E	42	SW.22.K	62	NE	82	SW.12.E
3	SW.20.postivecharger	23	SW.7.K	43	S	63	PSSM.162	83	SW.6.R
4	SW.19.postivecharger	24	SW.17.postivecharger	44	SW.5.K	64	SW.22.R	84	SW.23.K
5	SW.22.postivecharger	25	SW.18.negativecharger	45	SW.23.postivecharger	65	SW.17.R	85	SW.11.postivecharger
6	SW.9.postivecharger	26	SW.17.K	46	SW.17.E	66	SW.18.uncharger	86	SW.22.uncharger
7	SW.18.postivecharger	27	SW.21.uncharger	47	SW.10.K	67	SW.20.uncharger	87	P
8	K	28	SW.9.uncharger	48	SW.24.postivecharger	68	SW.13.D	88	SW.11.E
9	SW.13.negativecharger	29	SW.10.uncharger	49	E	69	SW.19.uncharger	89	BE.F412
10	SW.9.R	30	SW.18.postivecharger	50	SW.10.R	70	BE.F335	90	SW.15.uncharger
11	Q	31	SW.9.K	51	SW.24.uncharger	71	SW.19.R	91	SW.16.D
12	SW.20.K	32	SW.8.K	52	SW.15.D	72	SW.18.K	92	BE.F324
13	SW.14.E	33	SW.5.postivecharger	53	SW.14.postivecharger	73	BE.F292	93	QN
14	SW.6.postivecharger	34	SW.14.D	54	BE.F282	74	SW.6.K	94	SW.23.uncharger
15	SW.16.negativecharger	35	SW.20.R	55	SW.13.postivecharger	75	SW.21.R	95	AN
16	SW.15.E	36	BE.F327	56	SW.8.R	76	PSSM.130	96	PSSM.308
17	SW.10.postivecharger	37	HN	57	SW.12.negativecharger	77	SW.4.K	97	SW.3.postivecharger
18	SW.17.negativecharger	38	R	58	PSSM.320	78	KK.gap4	98	SW.11.uncharger
19	SW.22.postivecharger	39	SW.18.R	59	PSSM.480	79	SW.17.D	99	PSSM.590
20	SW.21.K	40	BE.F287	60	SW.11.negativecharger	80	PSSM.268	100	ER.gap4

EGAAC	EAAC	BE	PSSM
AAPC	CKSAAP	AAC	

(c)

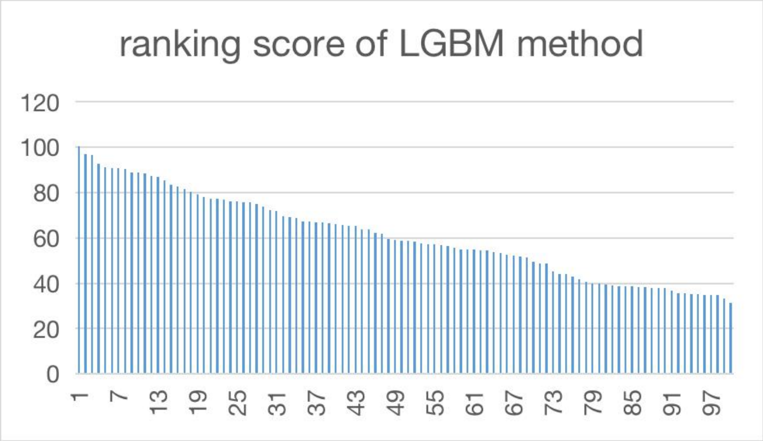

(b)

Figure s4: The statistics after LGBM feature selection method in the incorporated feature. After selection, it can be seen that EGAAC and EAAC consist the most part of the top-100 features, which is similar as the feature selection in Chi-square. (a) shows the composition of each types of feature after selection of incorporated features.(b) indicates the top-100 ranking score among the selection from incorporated feature.(c) listed the detailed top-100 features among incorporated feature

		Dimension	Dataset	Classifier	Sn	Sp	Acc	MCC	AUC
AAC	Original	20	Plant	libsvm	0.69	0.61	0.65	0.31	0.70
	MRMD	15	Plant	libsvm	0.71	0.66	0.68	0.39	0.74
AAPC	Original	400	Plant	libsvm	0.64	0.68	0.66	0.33	0.72
	MRMD	80	Plant	libsvm	0.70	0.66	0.68	0.40	0.76
BE	Original	620	Plant	libsvm	0.69	0.63	0.66	0.33	0.73
	MRMD	55	Plant	libsvm	0.69	0.66	0.68	0.40	0.76
CKSAAP	Original	1600	Plant	libsvm	0.65	0.67	0.66	0.32	0.73
	MRMD	80	Plant	libsvm	0.68	0.68	0.68	0.33	0.77
EAAC	Original	540	Plant	libsvm	0.68	0.72	0.71	0.40	0.78
	MRMD	100	Plant	libsvm	0.70	0.72	0.71	0.40	0.79
EGAAC	Original	135	Plant	libsvm	0.74	0.66	0.70	0.40	0.76
	MRMD	93	Plant	libsvm	0.76	0.70	0.73	0.41	0.78
PSSM	Original	620	Plant	libsvm	0.71	0.48	0.60	0.20	0.64
	MRMD	101	Plant	libsvm	0.74	0.71	0.73	0.41	0.76
AAC+AAPC+BE+CKSAAP+EAAC+EGAAC+PSSM	Original	3935	Plant	libsvm	0.74	0.71	0.73	0.43	0.78
	MRMD	524	Plant	libsvm	0.73	0.75	0.74	0.43	0.79

Table s5: Comparison of performance on the original feature and MRMD method selected feature with libsvm classifier. Here column “Dimension” shows the dimension of corresponding feature vectors before and after feature selection of each attribute. Slightly improvement can be seen in each attribute.

		Dimension	Dataset	Classifier	Sn	Sp	Acc	MCC	AUC
AAC	Original	20	Plant	libsvm	0.69	0.61	0.65	0.31	0.70
	MRMD	15	Plant	libsvm	0,74	0.72	0.73	0.40	0.78
AAPC	Original	400	Plant	libsvm	0.64	0.68	0.66	0.33	0.72
	MRMD	80	Plant	libsvm	0.70	0.71	0.71	0.40	0.78
BE	Original	620	Plant	libsvm	0.69	0.63	0.66	0.33	0.73
	MRMD	55	Plant	libsvm	0.67	0.71	0.69	0.40	0.76
CKSAAP	Original	1600	Plant	libsvm	0.65	0.67	0.66	0.32	0.73
	MRMD	80	Plant	libsvm	0.68	0.72	0.71	0.40	0.78
EAAC	Original	540	Plant	libsvm	0.68	0.72	0.71	0.40	0.78
	MRMD	100	Plant	libsvm	0.69	0.72	0.71	0.40	0.78
EGAAC	Original	135	Plant	libsvm	0.74	0.66	0.70	0.40	0.76
	MRMD	93	Plant	libsvm	0.70	0.72	0.71	0.40	0.78
PSSM	Original	620	Plant	libsvm	0.71	0.48	0.60	0.20	0.72
	MRMD	101	Plant	libsvm	0.69	0.60	0.65	0.40	0.78
AAC+AAPC+BE+CKSAAP+EAAC+EGAAC+PSSM	Original	3935	Plant	libsvm	0.74	0.71	0.73	0.43	0.78
	MRMD	524	Plant	libsvm	0.70	0.72	0.71	0.40	0.78

Table s6: Comparison of performance on the original feature and MRMD method selected feature with RF classifier. Here column “Dimension” shows the dimension of corresponding feature vectors before and after feature selection of each attribute. It can be seen that the improvement before and after the feature selection process is not as obvious as in the case of SVM classifier.

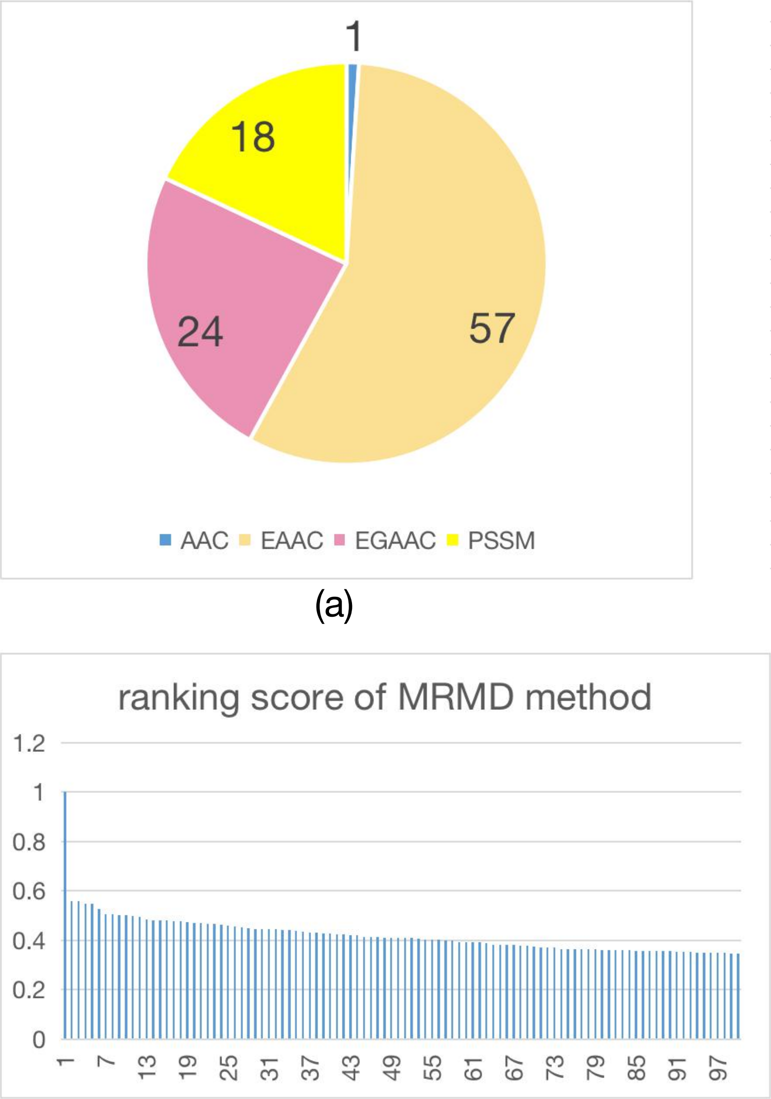

(a)

(b)

1	SW.24.Q	21	SW.16.L	41	SW.11.D	61	PSSM.58	81	PSSM.17
2	C	22	SW.20.D	42	SW.9.negativecharger	62	SW.11.T	82	SW.14.T
3	PSSM.38	23	SW.26.uncharger	43	SW.9.Q	63	SW.23.E	83	SW.17.T
4	SW.20.Q	24	SW.23.Q	44	SW.21.postivecharger	64	SW.10.Q	84	SW.27.C
5	SW.19.negativecharger	25	SW.10.D	45	SW.23.C	65	SW.23.F	85	PSSM.6
6	SW.13.Q	26	SW.15.Q	46	SW.9.postivecharger	66	SW.22.V	86	SW.9.aromatic
7	SW.25.Q	27	SW.23.negativecharger	47	SW.20.E	67	SW.1.postivecharger	87	SW.21.E
8	SW.3.negativecharger	28	PSSM.7	48	SW.23.D	68	SW.22.D	88	PSSM.85
9	SW.15.negativecharger	29	SW.14.Q	49	SW.17.L	69	PSSM.66	89	SW.12.T
10	SW.18.D	30	SW.9.D	50	SW.10.K	70	SW.17.D	90	SW.14.uncharger
11	SW.13.D	31	SW.26.D	51	SW.27.L	71	PSSM.87	91	PSSM.62
12	SW.19.D	32	PSSM.48	52	SW.22.P	72	SW.17.aromatic	92	SW.1.alphaticr
13	SW.21.Q	33	SW.16.Q	53	SW.5.postivecharger	73	SW.11.Q	93	SW.12.I
14	SW.12.Q	34	PSSM.68	54	SW.21.D	74	SW.16.C	94	PSSM.67
15	PSSM.18	35	SW.7.negativecharger	55	SW.27.negativecharger	75	SW.22.postivecharger	95	SW.6.negativecharger
16	SW.15.D	36	SW.17.Q	56	SW.9.T	76	SW.13.negativecharger	96	SW.12.N
17	SW.16.D	37	SW.27.Q	57	PSSM.98	77	SW.14.V	97	SW.12.aromatic
18	SW.25.D	38	SW.4.postivecharger	58	SW.12.P	78	SW.13.K	98	PSSM.36
19	PSSM.27	39	PSSM.78	59	SW.18.Q	79	SW.9.W	99	SW.17.E
20	SW.3.aromatic	40	SW.19.Q	60	SW.10.uncharger	80	SW.26.postivecharger	100	PSSM.88
	EGAAC		EAAC		BE		PSSM		
	AAPC		CKSAAP		AAC				

(c)

Figure s4: The statistics after MRMD feature selection method in the incorporated feature. After selection, it can be seen that EGAAC, EAAC and PSSM consist the most part of the top-100 features.(a) shows the composition of each types of feature after selection of incorporated features.(b) indicates the top-100 ranking score among the selection from incorporated feature.(c) listed the detailed top-100 features among incorporated feature

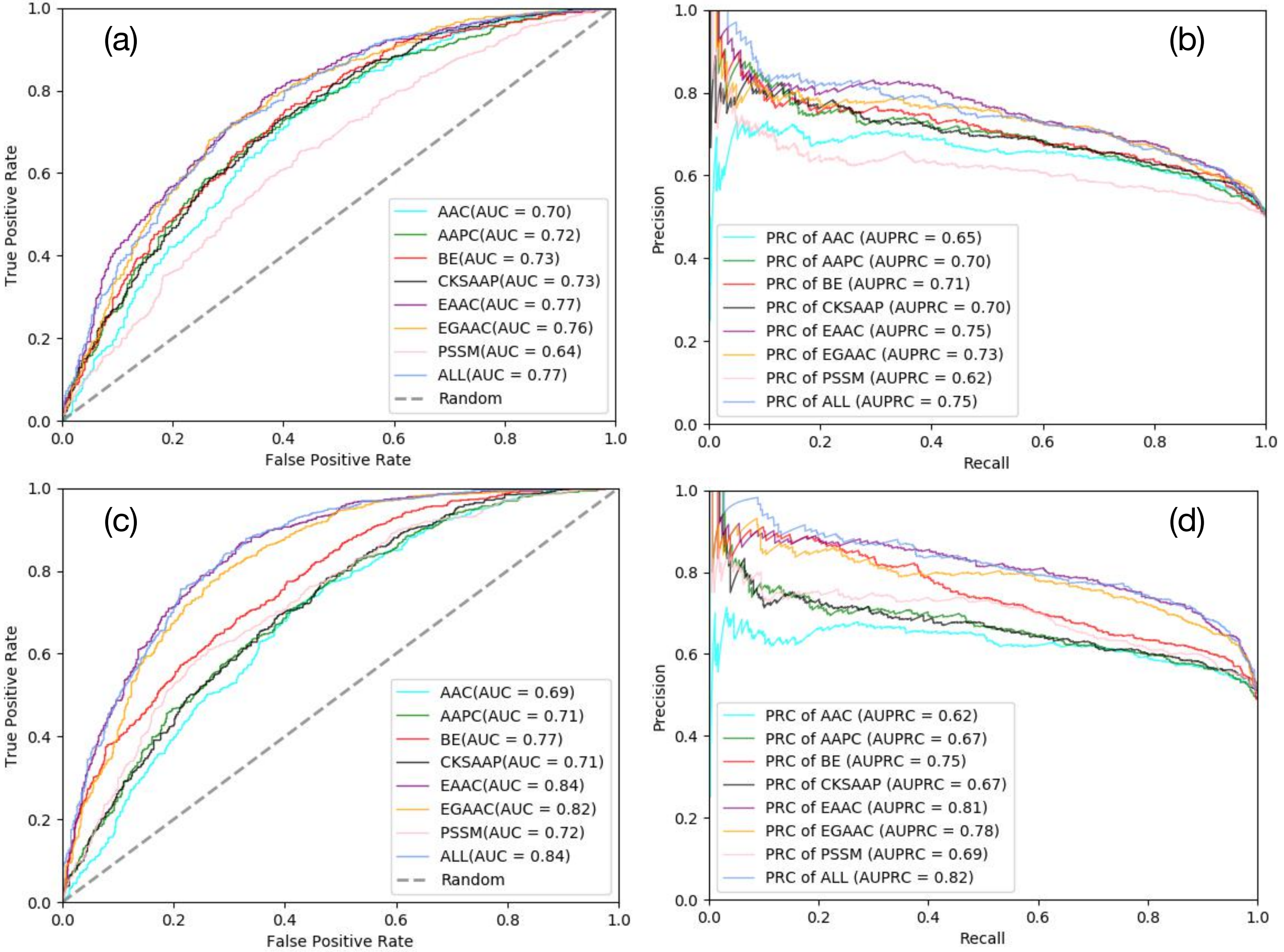

Figure s5: Comparison of ROC curves and PRC curves among SVM and random forest models trained with various attributes of plant dataset. (a): ROC curves trained by SVM with different features; (b): PRC curves trained by SVM with different features; (c):ROC curves trained by random forest with different features; (d): PRC curves trained by random forest with different features.

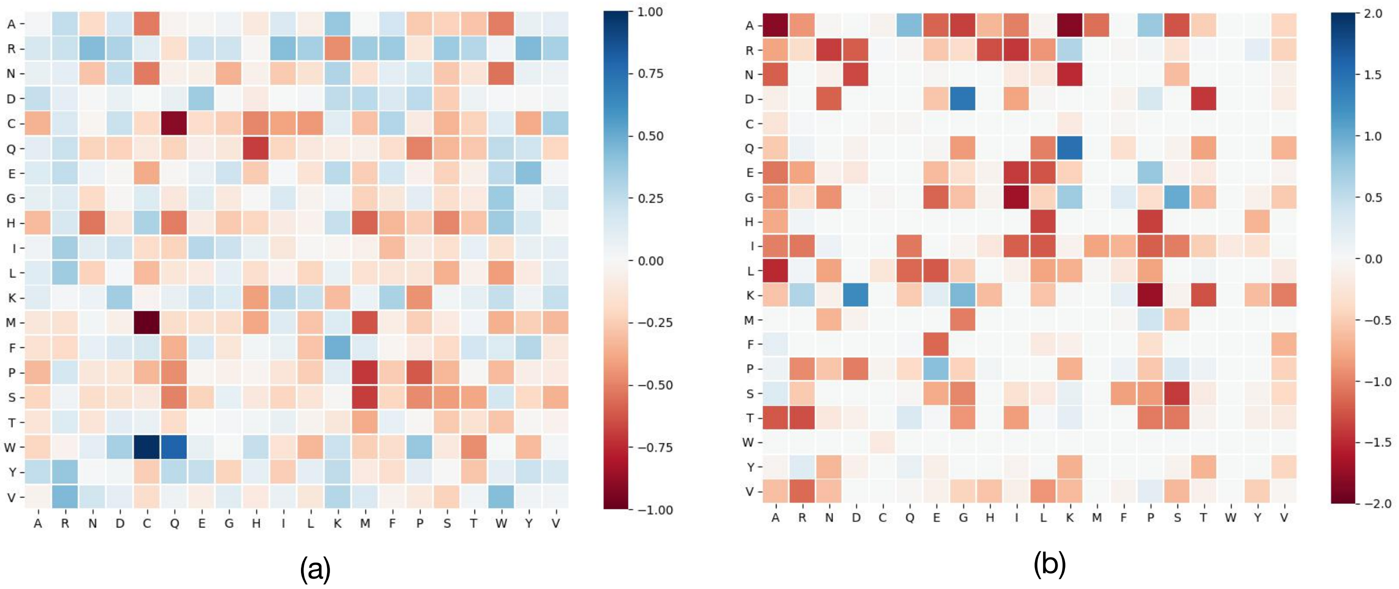

Figure s6: The statistics of each amino acid pair composition (AAPC) in (a) plant dataset and (b) mammalian dataset. From (a) it can be seen that pairs QC and CM occurred most frequently in positive sequences while pair CW occurred most frequently in negative sequences in the plant dataset. Fig.(b) indicated that pairs AA and KA occurred most frequently in positive sequences while pair KQ occurred most frequently in negative sequences in the mammalian dataset.

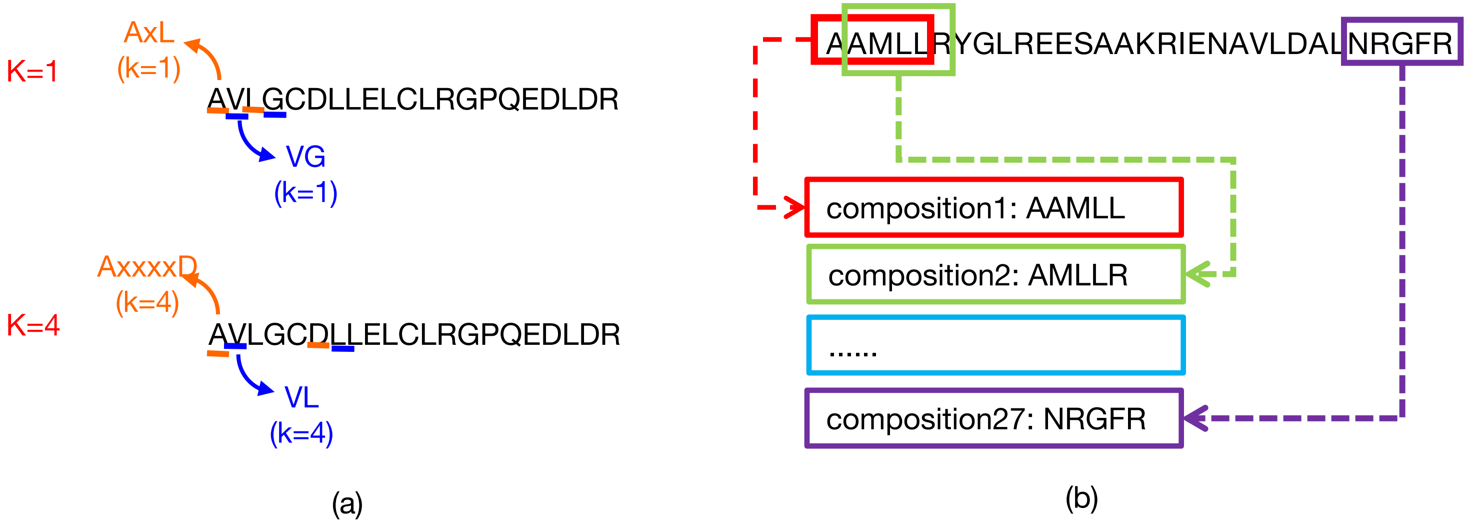

Figure s7: (a): illustration example of composition of K-spaced amino acid pairs. Here we have set k=4, where k = 1,2,3,4 are contained, hence totally there will be 4*400 = 1600 dimension for a feature vector in each single sequence; (b): illustration example of EAAC. Here window size of each composition is set as 5, the length of sequence equals 31, hence here the feature vector of EAAC will have 31-5 = 27 composition for any single sequence.

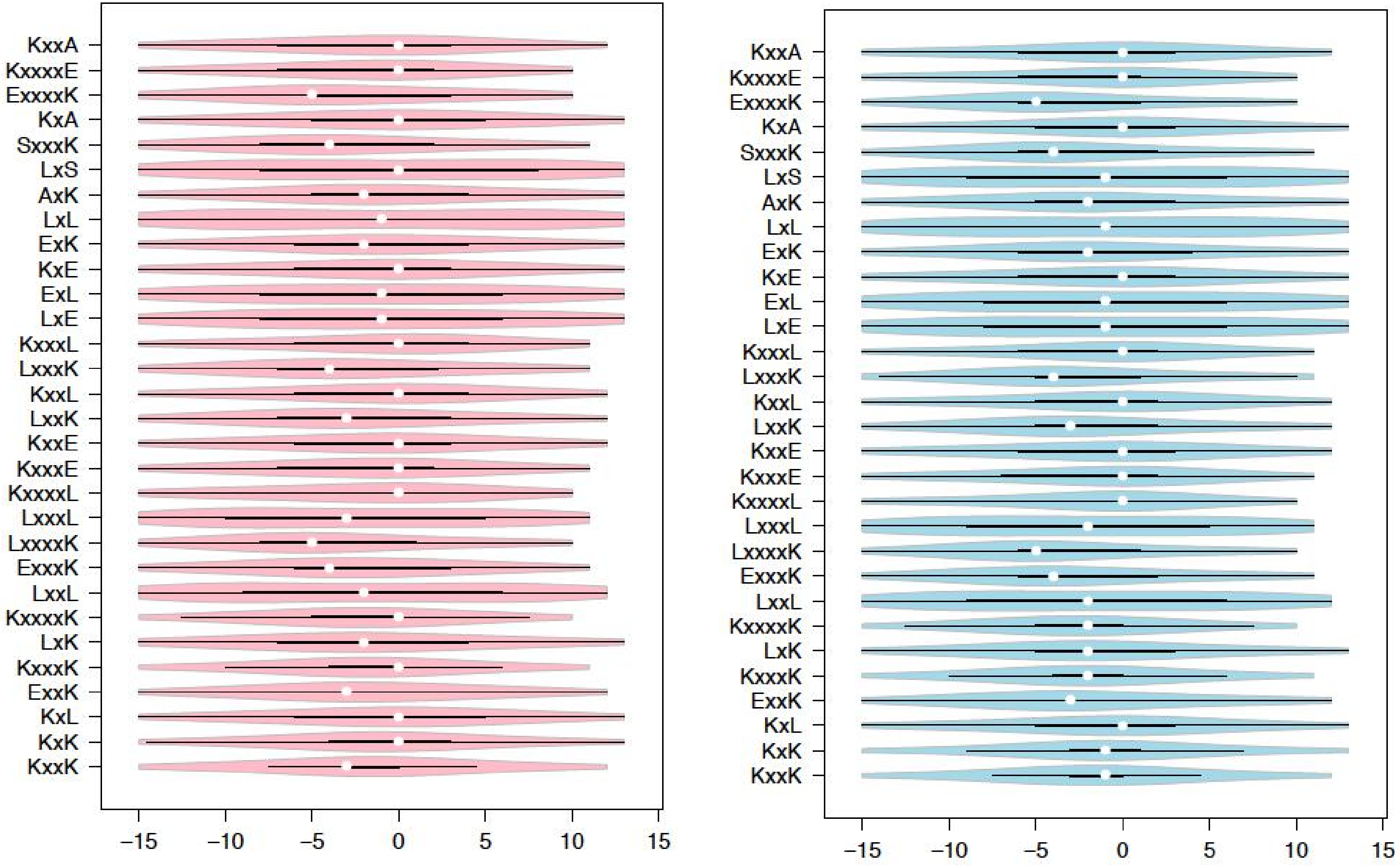

(a) (b)

Figure s8: Violin plot of CKSAAP of plant dataset. (a): positive samples; (b):negative samples. 30 pairs are listed here, in which the differences of occurrence in positive and negative are the largest, i.e.: the highest difference of occurrence between positive and negative shows in composition pair KxxK, the 30th largest differences shows in composition KxxA. The differences in the top4 pairs(KxxK, KxK, KxL and ExxK) in plant dataset are more obvious .

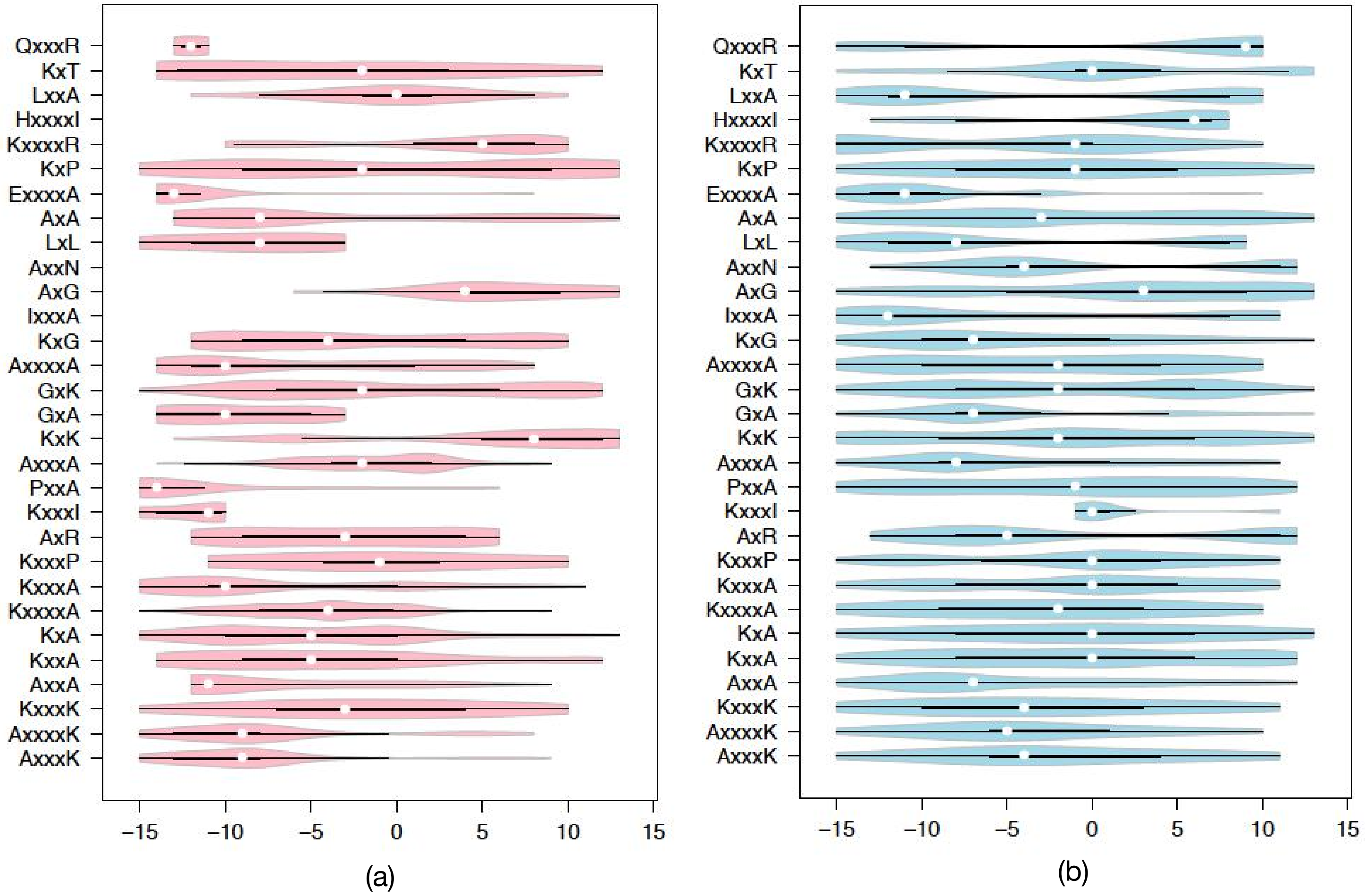

Figure s9: Violin plot of CKSAAP in (a)(b): positive and negative samples of mammalian dataset. 30 pairs are listed, in which the differences of occurrence in positive and negative are the largest, i.e.: in mammalian, the highest differences in occurrence between positive and negative shows in composition pair AxxxK, the 30th largest shows in QxxxR. In mammalian dataset it seems to have larger differences in positive and negative samples, as mentioned in AAC part, that is the main reason of better performances shown on the mammalian dataset than the plant dataset.

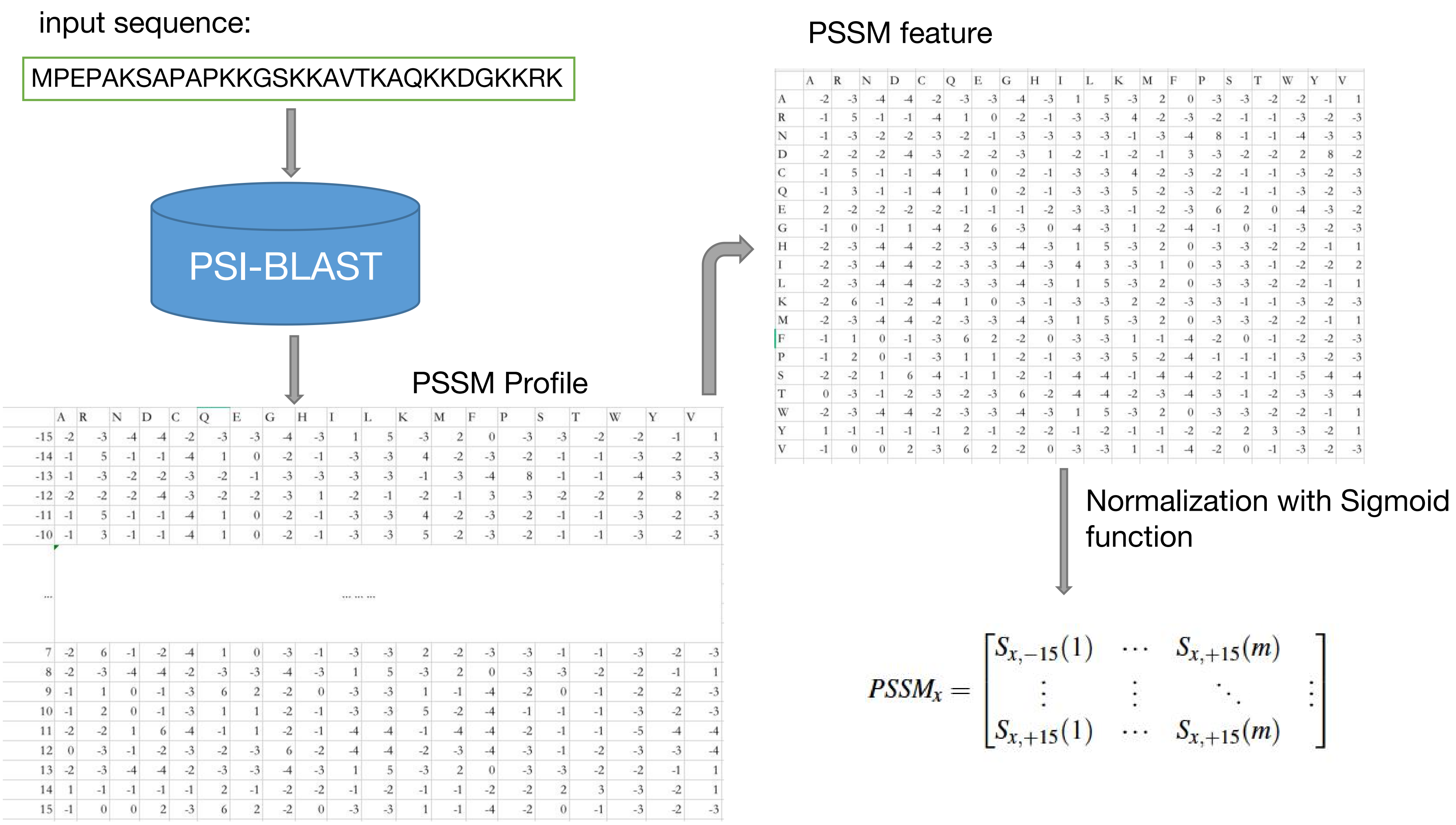

Figure s10: Workflow of generating PSSM feature matrix from PSI-BLAST.
